# Supplementary material for: Changing use of antidiabetic drugs in the UK: trends in prescribing 2000–2017
Source: BMJ Open. 2018 Jul 28;8(7):e022768. doi: 10.1136/bmjopen-2018-022768 (PMC6067400; doi:10.1136/bmjopen-2018-022768)
Supplement: Supplementary data [file bmjopen-2018-022768supp001.pdf]

Supplementary Table 1: "Other" drug initiations for 2000-2017: Top ten most common drug combinations.

Abbreviations: SU: Sulfonylurea, TZD: Thiazolidinediones, DPP4i: dipeptidyl peptidase 4 inhibitors, Glinides: Meglitinides

Drug combination	*Number of drugs prescribed at initiation*
Metformin and insulin	2,850
Metformin and TZD	1,405
TZD	1,393
Metformin, SU and TZD	908
DPP4i	902
Metformin and DPP4i	743
Metformin and TZD	708
SU and TZD	616
Metformin, SU and DPP4i	560
Glinides	440
Further drug combinations	3,463
Total	13,988

Supplementary Table 2 Proportion of drug classes prescribed for drug initiations for patients 2000-2017 (Percentage (95% CI)

Year	Metformin	SU	Metformin & SU	Insulin	Metformin & insulin	Other	Total
2000	41.29 (39.99, 42.59)	48.43 (47.21, 49.65)	4.04 (2.37, 5.71)	4.07 (2.4, 5.74)	0.37 (0, 2.07)	1.79 (0.11, 3.48)	13,262
2001	49.21 (48.06, 50.36)	39.94 (38.69, 41.18)	4.71 (3.14, 6.28)	3.63 (2.04, 5.21)	0.45 (0, 2.06)	2.07 (0.48, 3.67)	14,779
2002	57.68 (56.66, 58.71)	30.7 (29.39, 32.01)	5 (3.46, 6.53)	3.37 (1.82, 4.91)	0.59 (0, 2.16)	2.66 (1.11, 4.21)	15,531
2003	64.79 (63.88, 65.69)	23.36 (22.02, 24.69)	4.54 (3.05, 6.03)	3.49 (2, 4.99)	1.01 (0, 2.53)	2.81 (1.31, 4.31)	16,516
2004	68.51 (67.72, 69.31)	18.29 (17.01, 19.58)	4.77 (3.39, 6.15)	3.27 (1.88, 4.67)	0.93 (0, 2.34)	4.23 (2.84, 5.61)	19,121
2005	72.66 (71.9, 73.42)	15.19 (13.85, 16.54)	4.36 (2.94, 5.78)	2.63 (1.2, 4.07)	1.07 (0, 2.51)	4.08 (2.66, 5.51)	18,118
2006	74.17 (73.43, 74.91)	12.88 (11.52, 14.25)	4.52 (3.09, 5.94)	2.56 (1.12, 4.01)	1.28 (0, 2.74)	4.59 (3.16, 6.02)	17,940
2007	76.79 (76.11, 77.47)	10.62 (9.28, 11.96)	4.58 (3.19, 5.96)	2.54 (1.14, 3.94)	1.23 (0, 2.64)	4.24 (2.85, 5.62)	19,122
2008	78.91 (78.25, 79.57)	9.83 (8.47, 11.19)	4.63 (3.23, 6.03)	2.16 (0.74, 3.58)	1.24 (0, 2.66)	3.23 (1.82, 4.64)	18,651
2009	80.79 (80.17, 81.41)	8.64 (7.29, 9.99)	4.55 (3.17, 5.92)	1.87 (0.48, 3.27)	0.98 (0, 2.38)	3.17 (1.79, 4.56)	19,374
2010	80.7 (80.07, 81.32)	7.79 (6.44, 9.15)	4.68 (3.31, 6.06)	1.92 (0.53, 3.32)	1.23 (0, 2.63)	3.67 (2.29, 5.06)	19,275
2011	79.74 (79.08, 80.4)	7.69 (6.28, 9.11)	5.07 (3.63, 6.51)	1.91 (0.45, 3.37)	1.34 (0, 2.8)	4.25 (2.81, 5.7)	17,675
2012	79.45 (78.78, 80.13)	7.26 (5.83, 8.69)	5.53 (4.09, 6.98)	1.59 (0.12, 3.06)	1.17 (0, 2.65)	4.98 (3.54, 6.43)	17,458
2013	80.78 (80.1, 81.45)	6.18 (4.69, 7.68)	4.81 (3.31, 6.31)	1.73 (0.2, 3.26)	1.15 (0, 2.68)	5.35 (3.85, 6.85)	16,153
2014	80.28 (79.54, 81.03)	6.06 (4.44, 7.68)	4.84 (3.21, 6.47)	1.7 (0.04, 3.36)	1.17 (0, 2.84)	5.94 (4.32, 7.57)	13,715
2015	81.54 (80.78, 82.31)	5.7 (3.98, 7.43)	4.18 (2.44, 5.92)	1.47 (0, 3.23)	0.98 (0, 2.74)	6.13 (4.41, 7.85)	12,185
2016	84.02 (83.18, 84.87)	5.16 (3.1, 7.22)	3.32 (1.24, 5.4)	1 (0, 3.11)	0.77 (0, 2.88)	5.73 (3.68, 7.78)	8,588
2017	89.09 (87.86, 90.32)	5 (1.38, 8.63)	2.38 (0, 6.05)	0.58 (0, 4.28)	0.4 (0, 4.11)	2.56 (0, 6.23)	2,778

Supplementary Table 3: Proportion of drug classes prescribed for drug initiations for patients with eGFR ≤30ml/min/1.72m², 2000-2016 (Percentage (95% CI) **Notes***: 2017 data not provided as <10 people met the criteria between Jan-Jul 2017*

Year	*Metformin*	*SU*	*DPP4i*	*Insulin*	*Metformin & insulin*	*Metformin & SU*	*Other combinations*	*Total*
2000	29.09 (28.04, 30.14)	67.27 (65.67, 68.87)	0 (0, 0)	1.82 (1.56, 2.08)	0 (0, 0)	0 (0, 0)	1.82 (1.56, 2.08)	55
2001	26.92 (25.91, 27.93)	63.46 (61.91, 65.01)	0 (0, 0)	1.92 (1.65, 2.19)	0 (0, 0)	1.92 (1.65, 2.19)	5.77 (5.3, 6.24)	52
2002	23.38 (22.43, 24.33)	63.64 (62.08, 65.2)	0 (0, 0)	5.19 (4.74, 5.64)	0 (0, 0)	3.9 (3.51, 4.29)	3.9 (3.51, 4.29)	77
2003	34.52 (33.37, 35.67)	54.76 (53.31, 56.21)	0 (0, 0)	4.76 (4.33, 5.19)	1.19 (0.98, 1.4)	0 (0, 0)	4.76 (4.33, 5.19)	84
2004	25.89 (24.89, 26.89)	61.61 (60.08, 63.14)	0 (0, 0)	4.46 (4.05, 4.87)	0.89 (0.71, 1.07)	2.68 (2.36, 3)	4.46 (4.05, 4.87)	112
2005	28.41 (27.37, 29.45)	56.82 (55.35, 58.29)	0 (0, 0)	5.68 (5.21, 6.15)	0 (0, 0)	3.41 (3.05, 3.77)	5.68 (5.21, 6.15)	88
2006	31.52 (30.42, 32.62)	56.52 (55.05, 57.99)	0 (0, 0)	2.17 (1.88, 2.46)	0 (0, 0)	5.43 (4.97, 5.89)	4.35 (3.94, 4.76)	92
2007	30.67 (29.59, 31.75)	56 (54.54, 57.46)	0 (0, 0)	4 (3.61, 4.39)	0 (0, 0)	4 (3.61, 4.39)	5.33 (4.88, 5.78)	75
2008	29.49 (28.43, 30.55)	65.38 (63.8, 66.96)	1.28 (1.06, 1.5)	1.28 (1.06, 1.5)	0 (0, 0)	1.28 (1.06, 1.5)	1.28 (1.06, 1.5)	78
2009	27.06 (26.04, 28.08)	60 (58.49, 61.51)	1.18 (0.97, 1.39)	2.35 (2.05, 2.65)	0 (0, 0)	2.35 (2.05, 2.65)	7.06 (6.54, 7.58)	85
2010	28.77 (27.72, 29.82)	54.79 (53.34, 56.24)	2.74 (2.42, 3.06)	2.74 (2.42, 3.06)	2.74 (2.42, 3.06)	1.37 (1.14, 1.6)	6.85 (6.34, 7.36)	73
2011	20.75 (19.86, 21.64)	66.04 (64.46, 67.62)	0 (0, 0)	5.66 (5.19, 6.13)	0 (0, 0)	0 (0, 0)	7.55 (7.01, 8.09)	53
2012	25.53 (24.54, 26.52)	55.32 (53.87, 56.77)	4.26 (3.86, 4.66)	6.38 (5.89, 6.87)	0 (0, 0)	2.13 (1.84, 2.42)	6.38 (5.89, 6.87)	47
2013	23.4 (22.45, 24.35)	44.68 (43.38, 45.98)	21.28 (20.38, 22.18)	4.26 (3.86, 4.66)	0 (0, 0)	0 (0, 0)	6.38 (5.89, 6.87)	47
2014	17.65 (16.83, 18.47)	50.98 (49.59, 52.37)	17.65 (16.83, 18.47)	7.84 (7.29, 8.39)	0 (0, 0)	1.96 (1.69, 2.23)	3.92 (3.53, 4.31)	51
2015	8.11 (7.55, 8.67)	54.05 (52.62, 55.48)	29.73 (28.67, 30.79)	5.41 (4.95, 5.87)	0 (0, 0)	0 (0, 0)	2.7 (2.38, 3.02)	37
2016	9.52 (8.92, 10.12)	42.86 (41.59, 44.13)	33.33 (32.21, 34.45)	0 (0, 0)	0 (0, 0)	0 (0, 0)	14.29 (13.55, 15.03)	21

Supplementary Figure 1: *Drug initiations in people with reduced renal function, between 2000 and 2016*

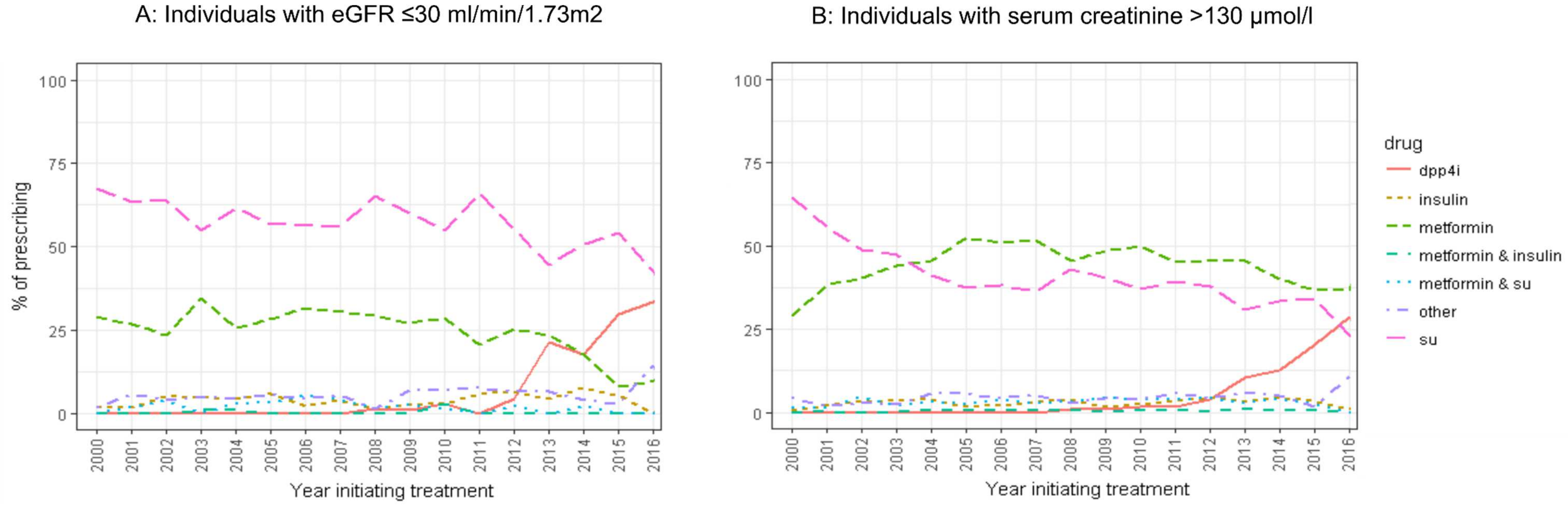

Supplementary Table 4: Numbers of prescriptions by year, drug prescribing at the first stage intensification, 2000-2017

Year	*DPP4i*	*SU*	*SGLT2i*	*TZD*	*Insulin*	*GLP1*	*Other*	*Total*
2000	0	86.83 (83.96, 89.7)	0	5.04 (0, 12.74)	4.55 (0, 12.27)	0	3.58 (0, 11.34)	***615***
2001	0	73.16 (70.51, 75.81)	0	17.3 (12.65, 21.95)	3.75 (0, 8.77)	0	5.79 (0.83, 10.76)	***1,468***
2002	0	64.76 (62.28, 67.25)	0	22.9 (19.22, 26.58)	3.56 (0, 7.68)	0	8.78 (4.77, 12.78)	***2,188***
2003	0	64.33 (62.25, 66.4)	0	28.4 (25.46, 31.34)	3.81 (0.4, 7.22)	0	3.46 (0.05, 6.88)	***3,176***
2004	0	55.3 (53.36, 57.23)	0	39.37 (37.12, 41.62)	3.55 (0.71, 6.38)	0	1.78 (0, 4.65)	***4,597***
2005	0	52.96 (51.08, 54.84)	0	43.18 (41.11, 45.25)	2.66 (0, 5.37)	0	1.19 (0, 3.92)	***5,111***
2006	0	51.29 (49.43, 53.15)	0	45.09 (43.12, 47.06)	2.53 (0, 5.16)	0	1.09 (0, 3.74)	***5,416***
2007	0.8 (0, 3.4)	59.17 (57.5, 60.84)	0	35.41 (33.31, 37.51)	3.2 (0.63, 5.77)	0.14 (0, 2.75)	1.28 (0, 3.88)	***5,623***
2008	4.94 (2.43, 7.44)	73.04 (71.71, 74.37)	0	17.19 (14.85, 19.52)	2.98 (0.45, 5.51)	0.6 (0, 3.16)	1.25 (0, 3.8)	***5,835***
2009	11.45 (9.24, 13.66)	70.65 (69.37, 71.92)	0	12.96 (10.77, 15.15)	2.36 (0.04, 4.68)	1.4 (0, 3.73)	1.19 (0, 3.53)	***6,953***
2010	21.38 (19.3, 23.45)	63.04 (61.62, 64.46)	0	10.39 (8.18, 12.61)	2.47 (0.15, 4.78)	2.04 (0, 4.35)	0.68 (0, 3.02)	***7,016***
2011	24.28 (22.14, 26.41)	63.75 (62.28, 65.23)	0	5.91 (3.53, 8.28)	2.88 (0.46, 5.3)	2.22 (0, 4.65)	0.96 (0, 3.4)	***6,384***
2012	29.76 (27.63, 31.89)	61.66 (60.08, 63.23)	0	2.5 (0, 5.01)	3.1 (0.6, 5.61)	2.18 (0, 4.69)	0.81 (0, 3.34)	***5,931***
2013	29.99 (27.77, 32.2)	61.09 (59.44, 62.74)	1.04 (0, 3.67)	2.24 (0, 4.86)	2.77 (0.16, 5.39)	1.77 (0, 4.39)	1.1 (0, 3.73)	***5,479***
2014	33.1 (30.79, 35.41)	54.09 (52.17, 56)	5.1 (2.35, 7.85)	1.76 (0, 4.56)	3.01 (0.23, 5.79)	1.97 (0, 4.76)	0.97 (0, 3.78)	***4,822***
2015	37.38 (35.1, 39.65)	45.1 (42.97, 47.23)	9.65 (6.91, 12.38)	1.94 (0, 4.79)	2.55 (0, 5.39)	2.33 (0, 5.18)	1.06 (0, 3.92)	***4,634***
2016	41.45 (38.92, 43.98)	35.36 (32.7, 38.02)	15.36 (12.31, 18.4)	2.51 (0, 5.77)	2.68 (0, 5.94)	1.77 (0, 5.05)	0.88 (0, 4.18)	***3,510***
2017	42.43 (38.11, 46.76)	30.01 (25.24, 34.78)	21.64 (16.6, 26.68)	2.28 (0, 7.92)	0.93 (0, 6.6)	1.78 (0, 7.42)	0.93 (0, 6.6)	***1,183***

Supplementary Table 5 *Mean days between starting metformin and starting intensification drug. Restricted to people intensifying treatment in 2016 (Mean (95% CI)*

	DPP4i	*SU*	*SGLT2i*	*TZD*	*Insulin*	*GLP1*
Mean days between starting metformin monotherapy and first stage of intensification	1345 (1288 to 1403)	1205 (1139 to 1270)	1117 (1033 to 1201)	1228 (1017 to 1439)	626 (444 to 809)	1174 (894 to 1455)

Supplementary Figure 2: Patterns of prescribing at the first stage of drug intensification across Clinical Commissioning Groups, 2014-2017 ***Notes:*** *Only CCGs with 10+ people intensifying treatment are included*

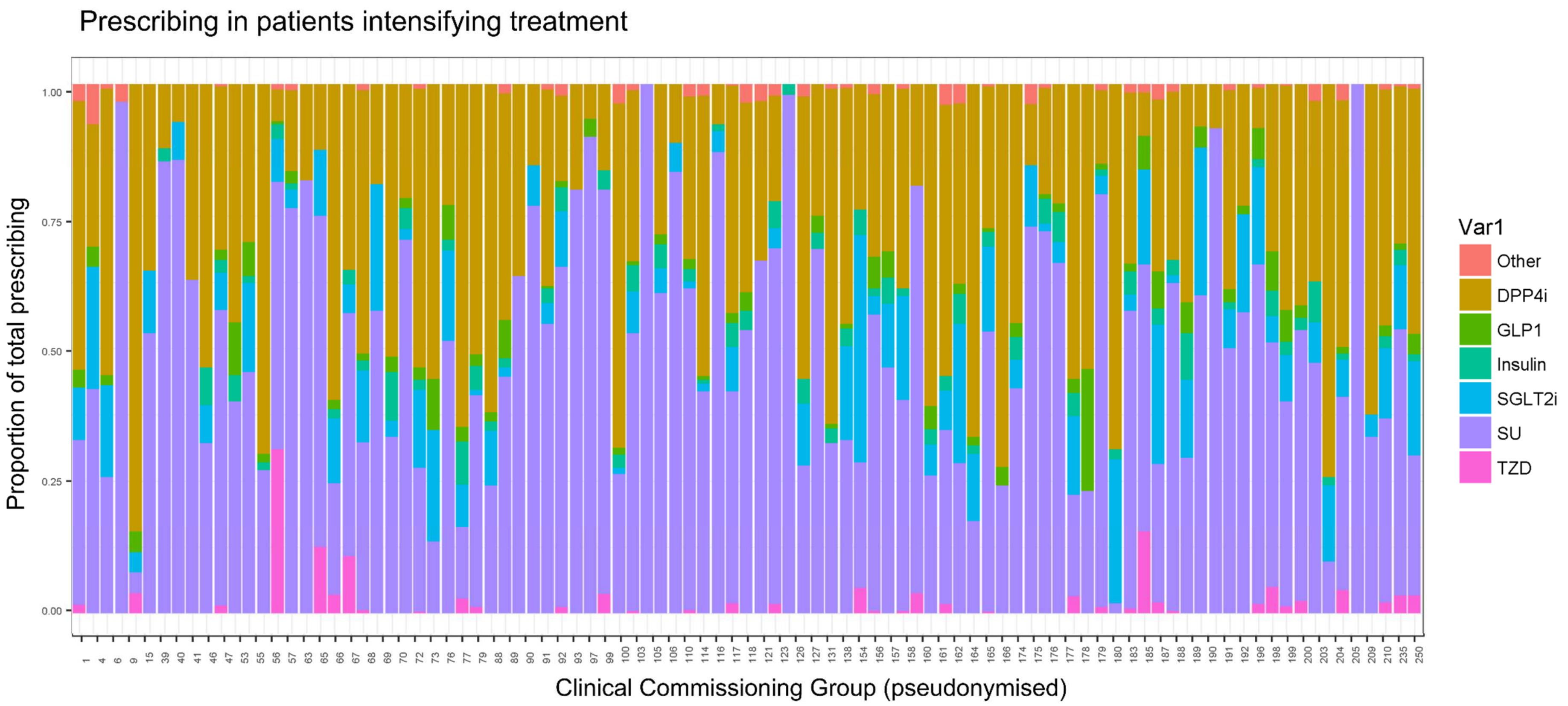

Supplementary Table 6: Percentage and 95% CI for drug prescriptions at the first stage of intensification by country, 2013-2017

Country	*SU*	*DPP4i*	*SGLT2i*	*Other combinations*	*Total*
England	47.86 (46.36, 49.36)	35.92 (34.26, 37.59)	8.64 (6.65, 10.62)	7.58 (5.58, 9.58)	*8,891*
N Ireland	26.47 (20.33, 32.62)	46.26 (41, 51.51)	17.78 (11.28, 24.28)	9.49 (2.67, 16.31)	*748*
Scotland	50.37 (47.08, 53.65)	29.71 (25.8, 33.62)	12 (7.62, 16.37)	7.92 (3.45, 12.4)	*1,767*
Wales	34.91 (31.78, 38.04)	44.8 (41.92, 47.68)	12.74 (9.12, 16.36)	7.54 (3.82, 11.27)	*2,558*

Drug code list: Diabetes Drugs

Product code	BNF code	Database build	BNF header	Product name
23	6010202	Feb-09	biguanides	metformin 500mg tablets
32	6010201	Feb-09	sulphonylureas	gliclazide 80mg tablets
93	6010202	Feb-09	biguanides	metformin 850mg tablets
322	0	Feb-09	unknown	humalog 100units/ml solution for injection 1.5ml cartridges (eli lilly and company ltd)
469	0	Feb-09	unknown	rosiglitazone 4mg tablets
479	6010203	Feb-09	other antidiabetic drugs	acarbose 50mg tablets
547	0	Feb-09	unknown	glipizide 2.5mg tablets
548	6010203	Feb-09	other antidiabetic drugs	pioglitazone 15mg tablets
735	6010202	Feb-09	biguanides	metformin 100mg/ml oral solution
1253	6010201	Feb-09	sulphonylureas	chlorpropamide 100mg tablets
1254	6010201	Feb-09	sulphonylureas	glibenclamide 5mg tablets
1587	0	Feb-09	unknown	monotard 100units/ml suspension for injection 10ml vials (novo nordisk ltd)
1588	06010101/06010300	Feb-09	short-acting insulins/diabetic ketoacidosis	actrapid 100iu/ml injection (novo nordisk ltd)
1592	06010101/06010300	Feb-09	short-acting insulins/diabetic ketoacidosis	actrapid penfill 100 100iu/ml penfill (novo nordisk ltd)
1593	6010102	Feb-09	intermediate- and long-acting insulins	insulatard penfill 100 100iu/ml penfill (novo nordisk ltd)
1594	0	Feb-09	unknown	actrapid novolet 100units/ml solution for injection (novo nordisk ltd)
1595	6010102	Feb-09	intermediate- and long-acting insulins	insulatard novolet 100units/ml suspension for injection (novo nordisk ltd)
1649	6010102	Feb-09	intermediate- and long-acting insulins	human actraphane 100iu/ml injection (novo nordisk ltd)
1805	6010102	Feb-09	intermediate- and long-acting insulins	mixtard 30/70 100unit/ml injection (novo nordisk ltd)
1806	6010102	Feb-09	intermediate- and long-acting insulins	penmix 30/70 100iu/ml penfill (novo nordisk ltd)
1840	06010101/06010300	Feb-09	short-acting insulins/diabetic ketoacidosis	humulin s 100unit/ml injection (eli lilly and company ltd)
1842	06010101/06010300	Feb-09	short-acting insulins/diabetic ketoacidosis	pork velosulin 100unit/ml injection (novo nordisk ltd)
1843	0	Feb-09	unknown	pork insulatard 100units/ml suspension for injection 10ml vials (novo nordisk ltd)
1844	0	Feb-09	unknown	ultratard 100units/ml suspension for injection 10ml vials (novo nordisk ltd)
1847	6010201	Feb-09	sulphonylureas	chlorpropamide 250mg tablets
1886	6010102	Feb-09	intermediate- and long-acting insulins	insulatard 100iu/ml ge injection (novo nordisk ltd)
1964	6010201	Feb-09	sulphonylureas	diamicron 80mg tablets (servier laboratories ltd)
1965	6010201	Feb-09	sulphonylureas	tolbutamide 500mg tablets
2219	6010201	Feb-09	sulphonylureas	glibenclamide 2.5mg tablets
2220	6010102	Feb-09	intermediate- and long-acting insulins	penmix 20/80 pen (novo nordisk ltd)
2221	0	Feb-09	unknown	mixtard 30 novolet 100units/ml suspension for injection (novo nordisk ltd)
2454	6010102	Feb-09	intermediate- and long-acting insulins	mixtard 30 penfill 100 100iu/ml penfill (novo nordisk ltd)
2455	0	Feb-09	unknown	mixtard 20 novolet 100units/ml suspension for injection (novo nordisk ltd)
2456	0	Feb-09	unknown	mixtard 10 novolet 100units/ml suspension for injection (novo nordisk ltd)
2459	0	Feb-09	unknown	pork mixtard 30 100units/ml suspension for injection 10ml vials (novo nordisk ltd)
2812	0	Feb-09	unknown	mixtard 40 novolet 100units/ml suspension for injection (novo nordisk ltd)
2928	0	Feb-09	unknown	metformin hcl 850 mg tab
2929	6010102	Feb-09	intermediate- and long-acting insulins	mixtard 30 100iu/ml ge injection (novo nordisk ltd)
3252	0	Feb-09	unknown	metformin hcl 500 mg tab
3396	6010102	Feb-09	intermediate- and long-acting insulins	penmix 10/90 penfill (novo nordisk ltd)
3439	6010102	Feb-09	intermediate- and long-acting insulins	penmix 10/90 pen (novo nordisk ltd)
3550	6010102	Feb-09	intermediate- and long-acting insulins	mixtard 40 penfill 100 100iu/ml penfill (novo nordisk ltd)
3551	6010102	Feb-09	intermediate- and long-acting insulins	mixtard 20 penfill 100 100iu/ml penfill (novo nordisk ltd)
3740	6010203	Feb-09	other antidiabetic drugs	guar gum 90% granules
4093	0	Feb-09	unknown	humulin m2 100units/ml suspension for injection 3ml cartridges (eli lilly and company ltd)
4129	0	Feb-09	unknown	insulin soluble porcine 100units/ml solution for injection 1.5ml cartridges
4163	6010102	Feb-09	intermediate- and long-acting insulins	rapitard mc 100unit/ml injection (novo nordisk ltd)
4198	6010102	Feb-09	intermediate- and long-acting insulins	humulin m3 100unit/ml m3 injection (eli lilly and company ltd)
4199	6010102	Feb-09	intermediate- and long-acting insulins	humulin m1 100unit/ml m1 injection (eli lilly and company ltd)
4247	0	Feb-09	unknown	insulin isophane porcine 100units/ml suspension for injection 1.5ml cartridges
4307	6010203	Feb-09	other antidiabetic drugs	guarina sachets (norgine pharmaceuticals ltd)
4426	0	Feb-09	unknown	chlorpropamide 500 mg tab
4706	0	Feb-09	unknown	velosulin 100units/ml solution for injection 10ml vials (novo nordisk ltd)
4715	6010102	Feb-09	intermediate- and long-acting insulins	humalog mix 25 25/75 100units/ml injection (eli lilly and company ltd)

4760	6010102	Feb-09	intermediate- and long-acting insulins	humulin i 100unit/ml injection (eli lilly and company ltd)
4784	6010102	Feb-09	intermediate- and long-acting insulins	lentard mc 100unit/ml injection (novo nordisk ltd)
4790	6010102	Feb-09	intermediate- and long-acting insulins	mixtard 50 penfill 100 100iu/ml penfill (novo nordisk ltd)
4862	6010201	Feb-09	sulphonylureas	diabetamide 2.5mg tablets (ashbourne pharmaceuticals ltd)
5021	6010101	Feb-09	short-acting insulins	novorapid penfill 100units/ml solution for injection 3ml cartridges (novo nordisk ltd)
5174	6010203	Feb-09	other antidiabetic drugs	acarbose 100mg tablets
5214	0	Feb-09	unknown	insulin lispro 100units/ml solution for injection 1.5ml cartridges
5227	0	Feb-09	unknown	rosiglitazone 8mg tablets
5250	6010102	Feb-09	intermediate- and long-acting insulins	insulin biphasic lispro human prb 25:75; 100 units/ml injection
5255	6010102	Feb-09	intermediate- and long-acting insulins	mixtard 10 penfill 100 100iu/ml penfill (novo nordisk ltd)
5276	6010201	Feb-09	sulphonylureas	glimepiride 1mg tablets
5316	6010201	Feb-09	sulphonylureas	glimepiride 4mg tablets
5353	6010201	Feb-09	sulphonylureas	glimepiride 2mg tablets
5501	6010102	Feb-09	intermediate- and long-acting insulins	insuman basal 100iu/ml injection (aventis pharma)
5621	6010203	Feb-09	other antidiabetic drugs	glucobay 50mg tablets (bayer plc)
5627	6010201	Feb-09	sulphonylureas	gliclazide 30mg modified-release tablets
5636	6010201	Feb-09	sulphonylureas	glipizide 5mg tablets
5678	6010203	Feb-09	other antidiabetic drugs	nateglinide 120mg tablets
5845	6010151	Feb-09	biphasic insulins	mixtard 30 innolet 100units/ml suspension for injection 3ml pre-filled pen (novo nordisk ltd)
5891	6010102	Feb-09	intermediate- and long-acting insulins	insulatard flexpen 100units/ml suspension for injection (novo nordisk ltd)
5892	6010101	Feb-09	short-acting insulins	novorapid flexpen 100units/ml solution for injection 3ml pre-filled pen (novo nordisk ltd)
5933	0	Feb-09	unknown	mixtard 50 novolet 100units/ml suspension for injection (novo nordisk ltd)
5953	6010102	Feb-09	intermediate- and long-acting insulins	insulin glargine 100iu/ml injection
5989	6010203	Feb-09	other antidiabetic drugs	nateglinide 180mg tablets
6057	6010102	Feb-09	intermediate- and long-acting insulins	lantus 100iu/ml injection (aventis pharma)
6061	6010102	Feb-09	intermediate- and long-acting insulins	novomix 30 30/70 100units/ml injection (novo nordisk ltd)
6209	6010101	Feb-09	short-acting insulins	novorapid 100units/ml solution for injection 10ml vials (novo nordisk ltd)
6337	6010201	Feb-09	sulphonylureas	glimepiride 3mg tablets
6447	6010101	Feb-09	short-acting insulins	insulin aspart human pyr 100 iu/ml injection
6855	0	Feb-09	unknown	avandamet 2mg/500mg tablets (glaxosmithkline uk ltd)
6958	6010102	Feb-09	intermediate- and long-acting insulins	levemir flexpen 100units/ml solution for injection 3ml pre-filled pen (novo nordisk ltd)
6965	6010102	Feb-09	intermediate- and long-acting insulins	levemir penfill 100units/ml solution for injection 3ml cartridges (novo nordisk ltd)
7048	6010202	Feb-09	biguanides	metformin 500mg modified-release tablets
7166	6010202	Feb-09	biguanides	glucophage 500mg tablets (merck serono ltd)
7228	6010151	Feb-09	biphasic insulins	novomix 30 flexpen 100units/ml suspension for injection 3ml pre-filled pen (novo nordisk ltd)
7231	6010151	Feb-09	biphasic insulins	mixtard 30 penfill 100units/ml suspension for injection 3ml cartridges (novo nordisk ltd)
7237	6010102	Feb-09	intermediate- and long-acting insulins	lantus 100units/ml solution for injection 3ml pre-filled optiset pen (sanofi)
7266	6010102	Feb-09	intermediate- and long-acting insulins	lantus 100units/ml solution for injection 3ml cartridges (sanofi)
7267	6010151	Feb-09	biphasic insulins	novomix 30 penfill 100units/ml suspension for injection 3ml cartridges (novo nordisk ltd)
7284	6010201	Feb-09	sulphonylureas	amaryl 2mg tablets (zentiva)
7300	6010151	Feb-09	biphasic insulins	mixtard 30 100units/ml suspension for injection 10ml vials (novo nordisk ltd)
7318	6010101	Feb-09	short-acting insulins	humalog 100units/ml solution for injection 3ml cartridges (eli lilly and company ltd)
7319	0	Feb-09	unknown	mixtard 20 penfill 100units/ml suspension for injection 3ml cartridges (novo nordisk ltd)
7325	0	Feb-09	unknown	avandamet 4mg/1000mg tablets (glaxosmithkline uk ltd)
7332	6010201	Feb-09	sulphonylureas	amaryl 1mg tablets (zentiva)
7349	6010101	Feb-09	short-acting insulins	actrapid 100units/ml solution for injection 10ml vials (novo nordisk ltd)
7350	6010102	Feb-09	intermediate- and long-acting insulins	insulin isophane porcine 100units/ml suspension for injection 10ml vials
7375	0	Feb-09	unknown	rosiglitazone 4mg / metformin 1g tablets
7393	6010102	Feb-09	intermediate- and long-acting insulins	insulin glargine 100units/ml solution for injection 3ml cartridges
7400	6010102	Feb-09	intermediate- and long-acting insulins	insulin glargine 100units/ml solution for injection 3ml pre-filled disposable devices
7402	6010102	Feb-09	intermediate- and long-acting insulins	lantus 100units/ml solution for injection 10ml vials (sanofi)
7409	6010201	Feb-09	sulphonylureas	amaryl 3mg tablets (zentiva)
7537	0	Feb-09	unknown	humulin zn 100units/ml suspension for injection 10ml vials (eli lilly and company ltd)
7610	6010202	Feb-09	biguanides	glucophage 850mg tablets (merck serono ltd)
7695	6010203	Feb-09	other antidiabetic drugs	guarem sachets (shire pharmaceuticals ltd)
7744	6010201	Feb-09	sulphonylureas	daonil 5mg tablets (sanofi)

7745	0	Feb-09	unknown	guar gum tab
7771	6010102	Feb-09	intermediate- and long-acting insulins	human protaphane penfill 100 100unit/ml penfill (novo nordisk ltd)
7772	6010102	Feb-09	intermediate- and long-acting insulins	human protaphane 100unit/ml injection (novo nordisk ltd)
7793	0	Feb-09	unknown	humaject m3 pen 100units/ml suspension for injection (eli lilly and company ltd)
7815	0	Feb-09	unknown	metformin 800 mg tab
7912	6010201	Feb-09	sulphonylureas	semi-daonil 2.5mg tablets (sanofi)
8034	6010201	Feb-09	sulphonylureas	diabinese 100mg tablet (pfizer ltd)
8118	6010102	Feb-09	intermediate- and long-acting insulins	humaject i 100iu/ml pen (eli lilly and company ltd)
8168	6010201	Feb-09	sulphonylureas	diabinese 250mg tablet (pfizer ltd)
8203	6010102	Feb-09	intermediate- and long-acting insulins	penmix 50/50 100iu/ml penfill (novo nordisk ltd)
8322	6010102	Feb-09	intermediate- and long-acting insulins	insulin zinc suspension mixed human pyr 100unit/ml injection
8390	0	Feb-09	unknown	gliquidone 30mg tablets
8841	0	Feb-09	unknown	humulin m5 100units/ml suspension for injection 10ml vials (eli lilly and company ltd)
8895	6010102	Feb-09	intermediate- and long-acting insulins	initard 50/50 100unit/ml injection (novo nordisk ltd)
8976	6010201	Feb-09	sulphonylureas	euglucon 2.5mg tablets (aventis pharma)
9105	6010203	Feb-09	other antidiabetic drugs	glucobay 100mg tablets (bayer plc)
9108	0	Feb-09	unknown	tolbutamide 250 mg tab
9341	6010102	Feb-09	intermediate- and long-acting insulins	insulin biphasic isophane human prb 30:70; 100 units/ml injection
9376	6010102	Feb-09	intermediate- and long-acting insulins	insulin zinc suspension crystalline human pyr 100unit/ml long acting injection
9503	6010102	Feb-09	intermediate- and long-acting insulins	hypurin bovine protamine zinc 100units/ml suspension for injection 10ml vials (wockhardt uk ltd)
9521	6010101	Feb-09	short-acting insulins	pork actrapid 100units/ml solution for injection 10ml vials (novo nordisk ltd)
9565	0	Feb-09	unknown	humaject s pen 100units/ml solution for injection (eli lilly and company ltd)
9618	0	Feb-09	unknown	hypurin porcine 30/70 mix 100units/ml suspension for injection 1.5ml cartridges (c p pharmaceuticals ltd)
9662	0	Feb-09	unknown	avandia 4mg tablets (glaxosmithkline uk ltd)
9699	6010203	Feb-09	other antidiabetic drugs	pioglitazone 30mg tablets
9707	6010203	Feb-09	other antidiabetic drugs	repaglinide 1mg tablets
9737	6010102	Feb-09	intermediate- and long-acting insulins	insulatard innolet 100iu/ml injection (novo nordisk ltd)
9748	6010203	Feb-09	other antidiabetic drugs	repaglinide 2mg tablets
9865	6010203	Feb-09	other antidiabetic drugs	repaglinide 500microgram tablets
10001	6010102	Feb-09	intermediate- and long-acting insulins	humalog mix50 pen 100units/ml suspension for injection 3ml pre-filled pen (eli lilly and company ltd)
10051	6010203	Feb-09	other antidiabetic drugs	pioglitazone 45mg tablets
10067	6010102	Feb-09	intermediate- and long-acting insulins	insulin biphasic aspart human pyr 30:70; 100 units/ml injection
10175	0	Feb-09	unknown	insulin isophane human 100units/ml suspension for injection 1.5ml cartridges
10184	6010102	Feb-09	intermediate- and long-acting insulins	insulin detemir 100 iu/ml solution for injection
10207	6010102	Feb-09	intermediate- and long-acting insulins	insulin isophane human 100units/ml suspension for injection 3ml cartridges
10208	6010102	Feb-09	intermediate- and long-acting insulins	insulatard innolet 100units/ml suspension for injection 3ml pre-filled pen (novo nordisk ltd)
10225	6010102	Feb-09	intermediate- and long-acting insulins	lantus 100units/ml solution for injection 3ml opticlik cartridges (sanofi)
10229	6010102	Feb-09	intermediate- and long-acting insulins	humulin i pen 100units/ml suspension for injection 3ml pre-filled pen (eli lilly and company ltd)
10243	6010102	Feb-09	intermediate- and long-acting insulins	humalog mix25 100units/ml suspension for injection 3ml cartridges (eli lilly and company ltd)
10244	0	Feb-09	unknown	mixtard 40 penfill 100units/ml suspension for injection 3ml cartridges (novo nordisk ltd)
10245	0	Feb-09	unknown	mixtard 10 penfill 100units/ml suspension for injection 3ml cartridges (novo nordisk ltd)
10259	6010102	Feb-09	intermediate- and long-acting insulins	insulin glargine 100units/ml solution for injection 10ml vials
10264	6010101	Feb-09	short-acting insulins	humalog pen 100units/ml solution for injection 3ml pre-filled pen (eli lilly and company ltd)
10277	6010151	Feb-09	biphasic insulins	humulin m3 100units/ml suspension for injection 3ml cartridges (eli lilly and company ltd)
10427	6010201	Feb-09	sulphonylureas	tolazamide 250mg tablet
10484	6010102	Feb-09	intermediate- and long-acting insulins	penmix 20/80 penfill (novo nordisk ltd)
10547	0	Feb-09	unknown	humulin lente 100units/ml suspension for injection 10ml vials (eli lilly and company ltd)
10572	06010101/06010300	Feb-09	short-acting insulins/diabetic ketoacidosis	insulin soluble bovine 100unit/ml injection
10887	6010102	Feb-09	intermediate- and long-acting insulins	penmix 40/60 100iu/ml penfill (novo nordisk ltd)
10910	6010102	Feb-09	intermediate- and long-acting insulins	humaject m2 100iu/ml m2 pen (eli lilly and company ltd)
10915	6010102	Feb-09	intermediate- and long-acting insulins	humaject m1 100iu/ml m1 pen (eli lilly and company ltd)
11055	6010102	Feb-09	intermediate- and long-acting insulins	insulin biphasic isophane human pyr 20:80; 100 units/ml injection
11056	6010102	Feb-09	intermediate- and long-acting insulins	insulin biphasic isophane human pyr 30:70; 100 units/ml injection
11080	6010102	Feb-09	intermediate- and long-acting insulins	insulin isophane human prb 100iu/ml injection

11107	6010102	Feb-09	intermediate- and long-acting insulins	humulin m4 100unit/ml m4 injection (eli lilly and company ltd)
11284	6010201	Feb-09	sulphonylureas	amaryl 4mg tablets (zentiva)
11316	6010203	Feb-09	other antidiabetic drugs	novonorm 500microgram tablets (novo nordisk ltd)
11321	6010203	Feb-09	other antidiabetic drugs	novonorm 1mg tablets (novo nordisk ltd)
11337	0	Feb-09	unknown	novorapid novolet 100units/ml solution for injection (novo nordisk ltd)
11366	6010203	Feb-09	other antidiabetic drugs	novonorm 2mg tablets (novo nordisk ltd)
11483	6010203	Feb-09	other antidiabetic drugs	nateglinide 60mg tablets
11601	0	Feb-09	unknown	rosiglitazone 2mg / metformin 500mg tablets
11604	0	Feb-09	unknown	rosiglitazone 1mg / metformin 500mg tablets
11609	06010202/06010203	Feb-09	biguanides/other antidiabetic drugs	metformin with rosiglitazone 500mg + 1mg tablet
11610	06010202/06010203	Feb-09	biguanides/other antidiabetic drugs	metformin with rosiglitazone 500mg + 2mg tablet
11695	6010201	Feb-09	sulphonylureas	diamicron 30mg mr tablets (servier laboratories ltd)
11717	0	Feb-09	unknown	rosiglitazone 2mg / metformin 1g tablets
11737	06010202/06010203	Feb-09	biguanides/other antidiabetic drugs	metformin with rosiglitazone 1000mg + 4mg tablet
11760	06010202/06010203	Feb-09	biguanides/other antidiabetic drugs	metformin with rosiglitazone 1000mg + 2mg tablet
11946	6010201	Feb-09	sulphonylureas	tolbutamide 50mg/ml injection
11990	6010202	Feb-09	biguanides	metformin 500mg/5ml oral solution sugar free
12035	6010102	Feb-09	intermediate- and long-acting insulins	insulin zinc mixed bovine 100units/ml suspension for injection 10ml vials
12245	6010201	Feb-09	sulphonylureas	glutril 25mg tablet (roche products ltd)
12259	6010201	Feb-09	sulphonylureas	glibornuride 25mg tablet
12297	06010101/06010300	Feb-09	short-acting insulins/diabetic ketoacidosis	hypurin bovine neutral 100unit/ml injection (c p pharmaceuticals ltd)
12299	6010102	Feb-09	intermediate- and long-acting insulins	semitard mc 100unit/ml injection (novo nordisk ltd)
12455	6010201	Feb-09	sulphonylureas	rastinon 500mg tablet (hoechst marion roussel)
12513	6010201	Feb-09	sulphonylureas	glibenese 5mg tablets (pfizer ltd)
12638	06010101/06010300	Feb-09	short-acting insulins/diabetic ketoacidosis	insulin soluble human pyr 100unit/ml injection
12654	06010101/06010300	Feb-09	short-acting insulins/diabetic ketoacidosis	insulin soluble human prb 100unit/ml injection
12818	0	Feb-09	unknown	human mixtard 50 100units/ml suspension for injection 10ml vials (novo nordisk ltd)
12897	0	Feb-09	unknown	guar gum 5g granules sachets sugar free
13277	0	Feb-09	unknown	mixtard 50 penfill 100units/ml suspension for injection 3ml cartridges (novo nordisk ltd)
13331	6010201	Feb-09	sulphonylureas	euglucon 5mg tablets (sanofi)
13416	6010102	Feb-09	intermediate- and long-acting insulins	insulin biphasic 100 units/ml injection
13516	6010102	Feb-09	intermediate- and long-acting insulins	hypurin bovine isophane 100unit/ml injection (c p pharmaceuticals ltd)
13622	06010101/06010300	Feb-09	short-acting insulins/diabetic ketoacidosis	hypurin porcine neutral 100unit/ml injection (c p pharmaceuticals ltd)
13628	6010203	Feb-09	other antidiabetic drugs	romozin 400mg tablet (glaxo wellcome uk ltd)
13729	6010102	Feb-09	intermediate- and long-acting insulins	insulin isophane human emp 100unit/ml injection
13819	0	Feb-09	unknown	hypurin porcine isophane 100units/ml suspension for injection 1.5ml cartridges (c p pharmaceuticals ltd)
13837	6010102	Feb-09	intermediate- and long-acting insulins	insulin biphasic isophane human prb 10:90; 100 units/ml injection
14164	0	Feb-09	unknown	avandamet 2mg/1000mg tablets (glaxosmithkline uk ltd)
14270	6010102	Feb-09	intermediate- and long-acting insulins	humalog mix25 pen 100units/ml suspension for injection 3ml pre-filled pen (eli lilly and company ltd)
14290	6010102	Feb-09	intermediate- and long-acting insulins	insulatard penfill 100units/ml suspension for injection 3ml cartridges (novo nordisk ltd)
14299	6010101	Feb-09	short-acting insulins	insulin glulisine 100units/ml solution for injection 3ml cartridges
14301	6010102	Feb-09	intermediate- and long-acting insulins	insulin detemir 100units/ml solution for injection 3ml cartridges
14313	6010101	Feb-09	short-acting insulins	insulin lispro 100units/ml solution for injection 3ml cartridges
14330	6010102	Feb-09	intermediate- and long-acting insulins	insulin detemir 100units/ml solution for injection 3ml pre-filled disposable devices
14339	06010101/06010300	Feb-09	short-acting insulins/diabetic ketoacidosis	hypurin bovine neutral 100units/ml solution for injection 10ml vials (wockhardt uk ltd)
14340	6010102	Feb-09	intermediate- and long-acting insulins	hypurin bovine isophane 100units/ml suspension for injection 10ml vials (wockhardt uk ltd)
14345	6010101	Feb-09	short-acting insulins	apidra 100units/ml solution for injection 3ml cartridges (sanofi)
14357	6010102	Feb-09	intermediate- and long-acting insulins	humulin i 100units/ml suspension for injection 3ml cartridges (eli lilly and company ltd)
14362	6010101	Feb-09	short-acting insulins	insulin lispro 100units/ml solution for injection 3ml pre-filled disposable devices
14505	6010102	Feb-09	intermediate- and long-acting insulins	insulin protamine zinc bovine 100units/ml suspension for injection 10ml vials
14619	0	Feb-09	unknown	insulin isophane biphasic porcine 30/70 100units/ml suspension for injection 1.5ml cartridges
14644	6010102	Feb-09	intermediate- and long-acting insulins	insulin biphasic isophane human prb 20:80; 100 units/ml injection
14649	6010102	Feb-09	intermediate- and long-acting insulins	insulin biphasic isophane human pyr 10:90; 100 units/ml injection
14918	6010102	Feb-09	intermediate- and long-acting insulins	humulin i 100units/ml suspension for injection 10ml vials (eli lilly and company ltd)
14925	6010102	Feb-09	intermediate- and long-acting insulins	insulin isophane human vial 100unit/ml sterile suspension injection
14928	6010102	Feb-09	intermediate- and long-acting insulins	insulatard 100units/ml suspension for injection 10ml vials (novo nordisk ltd)

14930	06010101/06010300	Feb-09	short-acting insulins/diabetic ketoacidosis	hypurin porcine neutral 100units/ml solution for injection 3ml cartridges (wockhardt uk ltd)
14933	6010102	Feb-09	intermediate- and long-acting insulins	hypurin porcine isophane 100units/ml suspension for injection 3ml cartridges (wockhardt uk ltd)
14938	06010101/06010300	Feb-09	short-acting insulins/diabetic ketoacidosis	insulin soluble bovine cartridge 100unit/ml solution for injection
14944	6010101	Feb-09	short-acting insulins	humulin s 100units/ml solution for injection 3ml cartridges (eli lilly and company ltd)
15199	6010102	Feb-09	intermediate- and long-acting insulins	insuman comb 25 100iu/ml injection (aventis pharma)
15232	0	Feb-09	unknown	avandia 8mg tablets (glaxosmithkline uk ltd)
15374	6010201	Feb-09	sulphonylureas	gliclazide 40mg/5ml oral suspension
15484	0	Feb-09	unknown	insulin isophane bovine 100units/ml suspension for injection 1.5ml cartridges
15710	06010101/06010300	Feb-09	short-acting insulins/diabetic ketoacidosis	insulin soluble human emp 100unit/ml injection
15955	6010203	Feb-09	other antidiabetic drugs	starlix 120mg tablets (novartis pharmaceuticals uk ltd)
15961	6010102	Feb-09	intermediate- and long-acting insulins	insulin isophane human crb 100iu/ml injection
16044	6010202	Feb-09	biguanides	glucophage sr 500mg tablets (merck serono ltd)
16129	6010101	Feb-09	short-acting insulins	insulin soluble human 100units/ml solution for injection 3ml cartridges
16142	6010101	Feb-09	short-acting insulins	insulin aspart 100units/ml solution for injection 3ml cartridges
16152	6010151	Feb-09	biphasic insulins	insulin isophane biphasic human 30/70 100units/ml suspension for injection 3ml cartridges
16160	0	Feb-09	unknown	humulin m3 pen 100units/ml suspension for injection 3ml pre-filled pen (eli lilly and company ltd)
16211	0	Feb-09	unknown	tolbutamide 100 mg tab
16213	0	Feb-09	unknown	metformin 250 mg tab
16602	6010201	Feb-09	sulphonylureas	calabren 2.5mg tablet (berk pharmaceuticals ltd)
16682	6010102	Feb-09	intermediate- and long-acting insulins	tempulin 100unit/ml injection (knoll ltd)
16700	6010102	Feb-09	intermediate- and long-acting insulins	insulin zinc mixed bovine vial 100unit/ml sterile suspension injection
17336	06010101/06010300	Feb-09	short-acting insulins/diabetic ketoacidosis	novopen 100unit/ml injection device (novo nordisk ltd)
17343	6010201	Feb-09	sulphonylureas	gliclazide 80mg tablets (a a h pharmaceuticals ltd)
17580	0	Feb-09	unknown	avandamet 1mg/500mg tablets (glaxosmithkline uk ltd)
17698	6010201	Feb-09	sulphonylureas	minodiab 5mg tablets (pfizer ltd)
17706	0	Feb-09	unknown	minodiab 2.5mg tablets (pfizer ltd)
17712	6010102	Feb-09	intermediate- and long-acting insulins	hypurin bovine lente 100units/ml suspension for injection 10ml vials (wockhardt uk ltd)
17731	6010102	Feb-09	intermediate- and long-acting insulins	penmix 50/50 100iu/ml injection (novo nordisk ltd)
17809	6010102	Feb-09	intermediate- and long-acting insulins	humaject m4 100iu/ml m4 pen (eli lilly and company ltd)
18220	6010203	Feb-09	other antidiabetic drugs	pioglitazone 15mg / metformin 850mg tablets
18224	6010101	Feb-09	short-acting insulins	humalog 100units/ml solution for injection 10ml vials (eli lilly and company ltd)
18461	0	Feb-09	unknown	insulin zinc mixed human 100units/ml suspension for injection 10ml vials
18590	6010102	Feb-09	intermediate- and long-acting insulins	insulin isophane bovine 100units/ml suspension for injection 10ml vials
18592	06010101/06010300	Feb-09	short-acting insulins/diabetic ketoacidosis	insulin soluble bovine 100units/ml solution for injection 10ml vials
18593	6010102	Feb-09	intermediate- and long-acting insulins	humalog mix50 100units/ml suspension for injection 3ml cartridges (eli lilly and company ltd)
18931	0	Feb-09	unknown	insulin zinc crystalline human 100units/ml suspension for injection 10ml vials
19336	6010201	Feb-09	sulphonylureas	tolazamide 100mg tablet
19472	6010203	Feb-09	other antidiabetic drugs	actos 45mg tablets (takeda uk ltd)
19491	6010101	Feb-09	short-acting insulins	apidra 100units/ml solution for injection 10ml vials (sanofi)
19513	6010151	Feb-09	biphasic insulins	humulin m3 100units/ml suspension for injection 10ml vials (eli lilly and company ltd)
19658	0	Feb-09	unknown	glurenorm 30mg tablets (sanofi)
19877	6010101	Feb-09	short-acting insulins	insulin aspart 100units/ml solution for injection 3ml pre-filled disposable devices
19878	6010151	Feb-09	biphasic insulins	insulin isophane biphasic human 30/70 100units/ml suspension for injection 3ml pre-filled disposable devices
20287	6010203	Feb-09	other antidiabetic drugs	actos 15mg tablets (takeda uk ltd)
20422	6010102	Feb-09	intermediate- and long-acting insulins	insuman comb 15 100iu/ml injection (aventis pharma)
20810	0	Feb-09	unknown	metformin
20889	6010203	Feb-09	other antidiabetic drugs	actos 30mg tablets (takeda uk ltd)
20995	6010151	Feb-09	biphasic insulins	hypurin porcine 30/70 mix 100units/ml suspension for injection 3ml cartridges (wockhardt uk ltd)
21110	6010102	Feb-09	intermediate- and long-acting insulins	insulin biphasic isophane human prb 50:50; 100 units/ml injection
21232	6010151	Feb-09	biphasic insulins	insulin isophane biphasic human 30/70 100units/ml suspension for injection 10ml vials
21235	6010101	Feb-09	short-acting insulins	humulin s 100units/ml solution for injection 10ml vials (eli lilly and company ltd)
21347	6010102	Feb-09	intermediate- and long-acting insulins	penmix 40/60 100iu/ml injection (novo nordisk ltd)
21374	6010102	Feb-09	intermediate- and long-acting insulins	insulin biphasic isophane human prb 40:60; 100 units/ml injection
21395	6010102	Feb-09	intermediate- and long-acting insulins	insulin biphasic isophane human pyr 40:60; 100 units/ml injection
21422	0	Feb-09	unknown	insulin isophane biphasic human 40/60 100units/ml suspension for injection 3ml cartridges

21424	6010201	Feb-09	sulphonylureas	glibenclamide 5mg/5ml oral suspension
21489	6010201	Feb-09	sulphonylureas	tolanase 250mg tablet (pharmacia ltd)
21554	6010102	Feb-09	intermediate- and long-acting insulins	insuman comb 50 100iu/ml injection (aventis pharma)
21564	6010201	Feb-09	sulphonylureas	gliclazide 80mg tablets (wockhardt uk ltd)
21583	6010101	Feb-09	short-acting insulins	apidra 100units/ml solution for injection 3ml pre-filled optiset pen (sanofi)
21590	6010101	Feb-09	short-acting insulins	insulin glulisine 100units/ml solution for injection 3ml pre-filled disposable devices
21832	6010201	Feb-09	sulphonylureas	diabetamide 5mg tablets (ashbourne pharmaceuticals ltd)
21870	0	Feb-09	unknown	rastinon
21892	6010201	Feb-09	sulphonylureas	diaglyk 80mg tablets (ashbourne pharmaceuticals ltd)
22058	6010102	Feb-09	intermediate- and long-acting insulins	pur-in mix 15/85 injection (c p pharmaceuticals ltd)
22145	6010201	Feb-09	sulphonylureas	tolanase 100mg tablet (pharmacia ltd)
22155	6010102	Feb-09	intermediate- and long-acting insulins	humaject m5 100iu/ml m5 pen (eli lilly and company ltd)
22614	0	Feb-09	unknown	daonil 10 mg tab
22636	0	Feb-09	unknown	tolbutamide 1 gm tab
22697	0	Feb-09	unknown	insulin isophane biphasic human 50/50 100units/ml suspension for injection 1.5ml cartridges
22858	6010201	Feb-09	sulphonylureas	acetohexamide 500mg tablets
22945	06010101/06010300	Feb-09	short-acting insulins/diabetic ketoacidosis	insuman rapid 100iu/ml injection (aventis pharma)
22983	6010101	Feb-09	short-acting insulins	insuman rapid 100units/ml solution for injection 3ml cartridges (sanofi)
23099	6010151	Feb-09	biphasic insulins	insulin aspart biphasic 30/70 100units/ml suspension for injection 3ml pre-filled disposable devices
23231	06010101/06010300	Feb-09	short-acting insulins/diabetic ketoacidosis	hypurin bovine neutral 100units/ml solution for injection 3ml cartridges (wockhardt uk ltd)
23945	6010203	Feb-09	other antidiabetic drugs	starlix 60mg tablets (novartis pharmaceuticals uk ltd)
23992	6010102	Feb-09	intermediate- and long-acting insulins	insuman basal 100units/ml suspension for injection 3ml pre-filled optiset pen (sanofi)
23993	6010101	Feb-09	short-acting insulins	insuman rapid 100units/ml solution for injection 3ml pre-filled optiset pen (sanofi)
24002	6010151	Feb-09	biphasic insulins	insuman comb 25 100units/ml suspension for injection 5ml vials (sanofi)
24593	06010101/06010300	Feb-09	short-acting insulins/diabetic ketoacidosis	neutral insulin bovine 100unit/ml injection
24795	6010151	Feb-09	biphasic insulins	insulin aspart biphasic 30/70 100units/ml suspension for injection 3ml cartridges
24800	6010151	Feb-09	biphasic insulins	hypurin porcine 30/70 mix 100units/ml suspension for injection 10ml vials (wockhardt uk ltd)
24846	06010101/06010300	Feb-09	short-acting insulins/diabetic ketoacidosis	pur-in neutral 100unit/ml injection (c p pharmaceuticals ltd)
24848	6010201	Feb-09	sulphonylureas	glymidine sodium 500mg tablet
24993	6010151	Feb-09	biphasic insulins	insuman comb 25 100units/ml suspension for injection 3ml cartridges (sanofi)
25133	6010151	Feb-09	biphasic insulins	insuman comb 25 100units/ml suspension for injection 3ml pre-filled optiset pen (sanofi)
25479	06010101/06010300	Feb-09	short-acting insulins/diabetic ketoacidosis	insulin soluble porcine 100units/ml solution for injection 3ml cartridges
25636	6010201	Feb-09	sulphonylureas	libanil 2.5mg tablet (approved prescription services ltd)
25639	0	Feb-09	unknown	guarem sachets
25678	6010202	Feb-09	biguanides	glucamet 500mg tablet (opus pharmaceuticals ltd)
25735	0	Feb-09	unknown	insulin isophane biphasic human 20/80 100units/ml suspension for injection 3ml cartridges
25736	0	Feb-09	unknown	insulin isophane biphasic human 10/90 100units/ml suspension for injection 3ml cartridges
25812	6010102	Feb-09	intermediate- and long-acting insulins	insulin isophane human 100units/ml suspension for injection 3ml pre-filled disposable devices
26060	6010101	Feb-09	short-acting insulins	insulin lispro 100units/ml solution for injection 10ml vials
26098	06010101/06010300	Feb-09	short-acting insulins/diabetic ketoacidosis	hypurin porcine neutral 100units/ml solution for injection 10ml vials (wockhardt uk ltd)
26118	6010201	Feb-09	sulphonylureas	dimelor 500mg tablet (eli lilly and company ltd)
26218	6010201	Feb-09	sulphonylureas	calabren 5mg tablet (berk pharmaceuticals ltd)
26258	6010202	Feb-09	biguanides	glucamet 850mg tablet (opus pharmaceuticals ltd)
26403	6010102	Feb-09	intermediate- and long-acting insulins	pur-in mix 25/75 injection (c p pharmaceuticals ltd)
26498	6010102	Feb-09	intermediate- and long-acting insulins	insulin zinc suspension mixed bovine and porcine 100unit/ml injection
26621	06010101/06010300	Feb-09	short-acting insulins/diabetic ketoacidosis	insulin soluble human crb 100iu/ml injection
27125	6010203	Feb-09	other antidiabetic drugs	starlix 180mg tablets (novartis pharmaceuticals uk ltd)
27177	6010102	Feb-09	intermediate- and long-acting insulins	insulin biphasic lispro human prb 50:50; 100 units/ml injection
27280	6010151	Feb-09	biphasic insulins	insulin isophane biphasic porcine 30/70 100units/ml suspension for injection 10ml vials
27396	06010101/06010300	Feb-09	short-acting insulins/diabetic ketoacidosis	insulin soluble porcine 100units/ml solution for injection 10ml vials
27402	6010101	Feb-09	short-acting insulins	insulin soluble human 100units/ml solution for injection 10ml vials
27461	6010102	Feb-09	intermediate- and long-acting insulins	insuman basal 100units/ml suspension for injection 3ml cartridges (sanofi)
27501	6010202	Feb-09	biguanides	orabet 500mg tablet (lagap)
27614	6010102	Feb-09	intermediate- and long-acting insulins	penmix 30/70 100iu/ml injection (novo nordisk ltd)

27969	6010201	Feb-09	sulphonylureas	glymese 250mg tablet (ddsa pharmaceuticals ltd)
28096	6010151	Feb-09	biphasic insulins	insulin isophane biphasic human 50/50 100units/ml suspension for injection 3ml cartridges
28101	6010101	Feb-09	short-acting insulins	insulin glulisine 100units/ml solution for injection 10ml vials
28183	6010102	Feb-09	intermediate- and long-acting insulins	hypurin porcine isophane 100units/ml suspension for injection 10ml vials (wockhardt uk ltd)
28185	6010102	Feb-09	intermediate- and long-acting insulins	insulin lispro biphasic 25/75 100units/ml suspension for injection 3ml cartridges
28442	6010101	Feb-09	short-acting insulins	insulin glulisine 100unit/ml solution for injection
28588	6010102	Feb-09	intermediate- and long-acting insulins	hypurin bovine isophane 100units/ml suspension for injection 3ml cartridges (wockhardt uk ltd)
28708	6010201	Feb-09	sulphonylureas	malix 2.5mg tablet (lagap)
29326	6010201	Feb-09	sulphonylureas	glipizide 5mg tablets (mylan ltd)
29567	6010101	Feb-09	short-acting insulins	insulin aspart 100units/ml solution for injection 10ml vials
29837	6010102	Feb-09	intermediate- and long-acting insulins	insulin biphasic isophane human prb 25:75; 100 units/ml injection
29939	6010201	Feb-09	sulphonylureas	gliclazide 80mg tablets (mylan ltd)
29953	6010101	Feb-09	short-acting insulins	apidra 100units/ml solution for injection 3ml opticlik cartridges (sanofi)
30209	06010101/06010300	Feb-09	short-acting insulins/diabetic ketoacidosis	actrapid mc 100unit/ml injection (arun products ltd)
30236	6010102	Feb-09	intermediate- and long-acting insulins	isophane insulin 100iu/ml injection
30316	06010202/06010203	Feb-09	biguanides/other antidiabetic drugs	metformin with pioglitazone 850mg + 15mg tablet
30460	6010201	Feb-09	sulphonylureas	malix 5mg tablet (lagap)
30686	6010102	Feb-09	intermediate- and long-acting insulins	insulin isophane porcine 100units/ml suspension for injection 3ml cartridges
30819	6010151	Feb-09	biphasic insulins	insuman comb 15 100units/ml suspension for injection 3ml pre-filled optiset pen (sanofi)
31077	6010203	Feb-09	other antidiabetic drugs	competact 15mg/850mg tablets (takeda uk ltd)
31146	6010202	Feb-09	biguanides	metsol 500mg/5ml oral solution (kappin ltd)
31205	6010151	Feb-09	biphasic insulins	insuman comb 50 100units/ml suspension for injection 3ml pre-filled optiset pen (sanofi)
31212	6010201	Feb-09	sulphonylureas	gliclazide 80mg tablets (actavis uk ltd)
31258	6010102	Feb-09	intermediate- and long-acting insulins	insulin lispro biphasic 25/75 100units/ml suspension for injection 3ml pre-filled disposable devices
31465	0	Feb-09	unknown	exubera 1mg inhalation powder blisters (pfizer ltd)
31467	0	Feb-09	unknown	exubera 3mg inhalation powder blisters (pfizer ltd)
31474	6010201	Feb-09	sulphonylureas	libanil 5mg tablet (approved prescription services ltd)
33087	6010202	Feb-09	biguanides	metformin 500mg tablets (actavis uk ltd)
33167	6010102	Feb-09	intermediate- and long-acting insulins	insulin biphasic isophane human crb 25:75; 100 units/ml injection
33232	0	Feb-09	unknown	insulin isophane biphasic human 50/50 100units/ml suspension for injection 5ml vials
33562	6010201	Feb-09	sulphonylureas	duclazide 80mg tablet (dumex ltd)
33673	6010201	Feb-09	sulphonylureas	tolbutamide 500mg tablets (actavis uk ltd)
33674	6010202	Feb-09	biguanides	metformin 850mg tablets (a a h pharmaceuticals ltd)
33966	6010102	Feb-09	intermediate- and long-acting insulins	insulatard 100unit/ml injection (novo nordisk ltd)
34004	6010202	Feb-09	biguanides	metformin 500mg tablets (ivax pharmaceuticals uk ltd)
34020	6010202	Feb-09	biguanides	metformin 850mg tablets (ivax pharmaceuticals uk ltd)
34031	6010102	Feb-09	intermediate- and long-acting insulins	monotard mc 100unit/ml injection (novo nordisk ltd)
34097	6010102	Feb-09	intermediate- and long-acting insulins	human initard 50/50 100unit/ml injection (novo nordisk ltd)
34135	6010202	Feb-09	biguanides	metformin 500mg tablet (m & a pharmachem ltd)
34323	6010202	Feb-09	biguanides	metformin 500mg tablets (a a h pharmaceuticals ltd)
34399	6010201	Feb-09	sulphonylureas	gliclazide 80mg tablets (ivax pharmaceuticals uk ltd)
34504	6010202	Feb-09	biguanides	metformin 500mg tablets (wockhardt uk ltd)
34507	6010201	Feb-09	sulphonylureas	glibenclamide 2.5mg tablets (wockhardt uk ltd)
34563	6010201	Feb-09	sulphonylureas	glibenclamide 5mg tablets (wockhardt uk ltd)
34598	6010202	Feb-09	biguanides	metformin 500mg tablets (mylan ltd)
34676	6010201	Feb-09	sulphonylureas	glibenclamide 2.5mg tablets (a a h pharmaceuticals ltd)
34697	6010202	Feb-09	biguanides	metformin 850mg tablets (wockhardt uk ltd)
34706	6010201	Feb-09	sulphonylureas	glibenclamide 2.5mg tablets (ivax pharmaceuticals uk ltd)
34742	6010202	Feb-09	biguanides	metformin 850mg tablets (teva uk ltd)
34802	6010201	Feb-09	sulphonylureas	glipizide 5mg tablets (ivax pharmaceuticals uk ltd)
34836	6010202	Feb-09	biguanides	metformin 850mg tablets (actavis uk ltd)
34917	6010202	Feb-09	biguanides	metformin 500mg tablets (teva uk ltd)
34932	6010201	Feb-09	sulphonylureas	gliclazide 80mg tablets (genus pharmaceuticals ltd)
34957	6010201	Feb-09	sulphonylureas	tolbutamide 500mg tablets (a a h pharmaceuticals ltd)
35022	6010203	Feb-09	other antidiabetic drugs	sitagliptin 100mg tablets
35144	6010203	Feb-09	other antidiabetic drugs	byetta 5micrograms/0.02ml solution for injection 1.2ml pre-filled disposable devices (astrazeneca uk ltd)

35149	6010203	Feb-09	other antidiabetic drugs	exenatide 10micrograms/0.04ml solution for injection 2.4ml pre-filled disposable devices
35150	6010203	Feb-09	other antidiabetic drugs	byetta 10micrograms/0.04ml solution for injection 2.4ml pre-filled disposable devices (astrazeneca uk ltd)
35251	6010203	Feb-09	other antidiabetic drugs	exenatide 5micrograms/0.02ml solution for injection 1.2ml pre-filled disposable devices
35253	6010151	Feb-09	biphasic insulins	insuman comb 50 100units/ml suspension for injection 3ml cartridges (sanofi)
35260	6010102	Feb-09	intermediate- and long-acting insulins	levemir innolet 100units/ml solution for injection 3ml pre-filled pen (novo nordisk ltd)
35462	6010203	Feb-09	other antidiabetic drugs	januvia 100mg tablets (merck sharp & dohme ltd)
35468	6010102	Feb-09	intermediate- and long-acting insulins	insuman basal 100units/ml suspension for injection 5ml vials (sanofi)
35561	6010203	Feb-09	other antidiabetic drugs	prandin 2mg tablets (novo nordisk ltd)
35701	6010102	Feb-09	intermediate- and long-acting insulins	insulin lispro biphasic 50/50 100units/ml suspension for injection 3ml pre-filled disposable devices
36031	6010151	Feb-09	biphasic insulins	insulin isophane biphasic porcine 30/70 100units/ml suspension for injection 3ml cartridges
36066	6010102	Feb-09	intermediate- and long-acting insulins	insulin isophane bovine 100units/ml suspension for injection 3ml cartridges
36146	6010102	Feb-09	intermediate- and long-acting insulins	insulin lispro biphasic 50/50 100units/ml suspension for injection 3ml cartridges
36194	6010151	Feb-09	biphasic insulins	insulin isophane biphasic human 25/75 100units/ml suspension for injection 3ml cartridges
36355	0	Feb-09	unknown	insulin human 1mg inhalation powder blisters
36356	0	Feb-09	unknown	insulin human 3mg inhalation powder blisters
36430	6010101	Feb-09	short-acting insulins	insulin soluble human 100units/ml solution for injection 3ml pre-filled disposable devices
36513	06010101/06010300	Feb-09	short-acting insulins/diabetic ketoacidosis	velosulin cartridge 100unit/ml injection (novo nordisk ltd)
36774	6010203	Feb-09	other antidiabetic drugs	prandin 1mg tablets (novo nordisk ltd)
36853	6010102	Feb-09	intermediate- and long-acting insulins	lantus 100units/ml solution for injection 3ml pre-filled solostar pen (sanofi)
36856	6010201	Feb-09	sulphonylureas	gliclazide 80mg tablets (sandoz ltd)
36920	6010101	Feb-09	short-acting insulins	apidra 100units/ml solution for injection 3ml pre-filled solostar pen (sanofi)
36948	6010203	Feb-09	other antidiabetic drugs	prandin 0.5mg tablets (novo nordisk ltd)
37617	6010203	Feb-09	other antidiabetic drugs	rosiglitazone 2mg tablet
37874	6010203	Feb-09	other antidiabetic drugs	vildagliptin 50mg / metformin 850mg tablets
37875	6010203	Feb-09	other antidiabetic drugs	vildagliptin 50mg tablets
37902	6010203	Feb-09	other antidiabetic drugs	vildagliptin 50mg / metformin 1g tablets
38355	6010202	Feb-09	biguanides	metformin 750mg modified-release tablets
38400	6010202	Feb-09	biguanides	glucophage sr 750mg tablets (merck serono ltd)
38422	6010102	Feb-09	intermediate- and long-acting insulins	isophane 100iu/ml injection (celltech pharma europe ltd)
38551	6010203	Feb-09	other antidiabetic drugs	eucreas 50mg/1000mg tablets (novartis pharmaceuticals uk ltd)
38986	6010101	Feb-09	short-acting insulins	humalog kwikpen 100units/ml solution for injection 3ml pre-filled pen (eli lilly and company ltd)
39006	6010102	Feb-09	intermediate- and long-acting insulins	humalog mix25 kwikpen 100units/ml suspension for injection 3ml pre-filled pen (eli lilly and company ltd)
39086	6010102	Apr-09	intermediate- and long-acting insulins	humalog mix50 kwikpen 100units/ml suspension for injection 3ml pre-filled pen (eli lilly and company ltd)
39149	6010203	Apr-09	other antidiabetic drugs	galvus 50mg tablets (novartis pharmaceuticals uk ltd)
39203	6010203	Apr-09	other antidiabetic drugs	eucreas 50mg/850mg tablets (novartis pharmaceuticals uk ltd)
39560	6010202	May-09	biguanides	bolamyn sr 500mg tablets (teva uk ltd)
39598	6010202	May-09	biguanides	metformin 1g modified-release tablets
39729	6010202	Jun-09	biguanides	glucophage sr 1000mg tablets (merck serono ltd)
39988	6010202	Aug-09	biguanides	metformin 500mg oral powder sachets sugar free
40007	6010202	Aug-09	biguanides	glucophage 1000mg oral powder sachets (merck serono ltd)
40110	6010202	Aug-09	biguanides	glucophage 500mg oral powder sachets (merck serono ltd)
40233	6010202	Aug-09	biguanides	metformin 1g oral powder sachets sugar free
40365	6010201	Sep-09	sulphonylureas	glimepiride 1mg tablets (actavis uk ltd)
40425	6010201	Sep-09	sulphonylureas	nazdol mr 30mg tablets (teva uk ltd)
40642	6010203	Oct-09	other antidiabetic drugs	victoza 6mg/ml solution for injection 3ml pre-filled pen (novo nordisk ltd)
40693	6010203	Nov-09	other antidiabetic drugs	liraglutide 6mg/ml solution for injection 3ml pre-filled disposable devices
41120	6010151	Jan-10	biphasic insulins	insulin isophane biphasic human 50/50 100units/ml suspension for injection 3ml pre-filled disposable devices
41204	6010203	Feb-10	other antidiabetic drugs	saxagliptin 5mg tablets
41431	6010203	Mar-10	other antidiabetic drugs	onglyza 5mg tablets (astrazeneca uk ltd)
41558	6010201	Mar-10	sulphonylureas	glibenclamide 5mg tablets (teva uk ltd)
41559	6010201	Mar-10	sulphonylureas	glibenclamide 5mg tablets (a a h pharmaceuticals ltd)
41593	6010201	Mar-10	sulphonylureas	glibenclamide 2.5mg tablets (teva uk ltd)
41834	6010102	Apr-10	intermediate- and long-acting insulins	insulin zinc suspension lente 100iu/ml injection (celltech pharma europe ltd)
41898	0	Apr-10	unknown	glibenclamide

41959	06010101/06010300	Apr-10	short-acting insulins/diabetic ketoacidosis	penject 100unit/ml injection device (hypoguard ltd)
42161	6010202	Apr-10	biguanides	orabet 500mg tablet (sandoz ltd)
42395	6010102	May-10	intermediate- and long-acting insulins	humalog mix25 100units/ml suspension for injection 10ml vials (eli lilly and company ltd)
42790	6010201	Jul-10	sulphonylureas	gliclazide 80mg tablet (merck generics (uk) ltd)
42954	6010151	Jul-10	biphasic insulins	insulin isophane biphasic human 25/75 100units/ml suspension for injection 5ml vials
43065	6010201	Aug-10	sulphonylureas	gliclazide 40mg tablets
43270	6010202	Aug-10	biguanides	metformin 500mg/5ml oral solution sugar free (rosemont pharmaceuticals ltd)
43465	6010201	Sep-10	sulphonylureas	zicron 40mg tablets (bristol laboratories ltd)
43619	6010203	Oct-10	other antidiabetic drugs	metformin 1g / sitagliptin 50mg tablets
43684	6010203	Oct-10	other antidiabetic drugs	janumet 50mg/1000mg tablets (merck sharp & dohme ltd)
43950	6010102	Nov-10	intermediate- and long-acting insulins	humulin i kwikpen 100units/ml suspension for injection 3ml pre-filled pen (eli lilly and company ltd)
43953	6010102	Nov-10	intermediate- and long-acting insulins	insulin lispro biphasic 25/75 100units/ml suspension for injection 10ml vials
43991	6010151	Dec-10	biphasic insulins	humulin m3 kwikpen 100units/ml suspension for injection 3ml pre-filled pen (eli lilly and company ltd)
44250	6010202	Jan-11	biguanides	metformin 500mg/5ml oral solution (hillcross pharmaceuticals ltd)
44251	6010102	Jan-11	intermediate- and long-acting insulins	insulin zinc suspension mixed porcine 100unit/ml injection
44304	6010201	Jan-11	sulphonylureas	glyconon 500mg tablet (ddsa pharmaceuticals ltd)
44378	6010151	Jan-11	biphasic insulins	insulin isophane biphasic human 25/75 100units/ml suspension for injection 3ml pre-filled disposable devices
44473	6010201	Jan-11	sulphonylureas	edicil mr 30mg tablets (teva uk ltd)
44480	6010151	Feb-11	biphasic insulins	insuman comb 25 100units/ml suspension for injection 3ml pre-filled solostar pen (sanofi)
44738	6010201	Mar-11	sulphonylureas	niddaryl 1mg tablets (dee pharmaceuticals ltd)
45158	6010151	May-11	biphasic insulins	insuman comb 15 100units/ml suspension for injection 3ml cartridges (sanofi)
45215	6010201	May-11	sulphonylureas	gliclazide 80mg tablet (neo laboratories ltd)
45581	6010202	Jun-11	biguanides	metabet sr 500mg tablets (morningside healthcare ltd)
45775	6010203	Aug-11	other antidiabetic drugs	saxagliptin 2.5mg tablets
45821	6010203	Aug-11	other antidiabetic drugs	onglyza 2.5mg tablets (astrazeneca uk ltd)
45831	6010201	Aug-11	sulphonylureas	dacadis mr 30mg tablets (mylan ltd)
46001	6010102	Aug-11	intermediate- and long-acting insulins	insuman basal 100units/ml suspension for injection 3ml pre-filled solostar pen (sanofi)
46458	6010203	Nov-11	other antidiabetic drugs	exenatide 2mg powder and solvent for prolonged-release suspension for injection vials
46469	6010203	Nov-11	other antidiabetic drugs	bydureon 2mg powder and solvent for prolonged-release suspension for injection vials (astrazeneca uk ltd)
46665	6010203	Jan-12	other antidiabetic drugs	linagliptin 5mg tablets
46666	6010101	Jan-12	short-acting insulins	novorapid flextouch 100units/ml solution for injection 3ml pre-filled pen (novo nordisk ltd)
46716	6010203	Jan-12	other antidiabetic drugs	trajenta 5mg tablets (boehringer ingelheim ltd)
46927	6010201	Mar-12	sulphonylureas	tolbutamide 500mg tablets (teva uk ltd)
46989	6010202	Mar-12	biguanides	metabet sr 1000mg tablets (morningside healthcare ltd)
47074	6010201	Apr-12	sulphonylureas	gliclazide 80mg/5ml oral suspension
47360	06010101/06010300	Jun-12	short-acting insulins/diabetic ketoacidosis	neutral insulin 100unit/ml injection (celltech pharma europe ltd)
47856	6010102	Oct-12	intermediate- and long-acting insulins	neuphane 100unit/ml injection (wellcome medical division)
47894	6010201	Oct-12	sulphonylureas	nazdol mr 30mg tablets (consilient health ltd)
47939	6010202	Oct-12	biguanides	glucient sr 500mg tablets (consilient health ltd)
48056	6010201	Nov-12	sulphonylureas	gliclazide 80mg tablets (sovereign medical ltd)
48120	6010203	Dec-12	other antidiabetic drugs	avandia 2mg tablet (glaxosmithkline uk ltd)
48139	6010203	Dec-12	other antidiabetic drugs	pioglitazone 30mg tablets (a a h pharmaceuticals ltd)
48149	6010202	Dec-12	biguanides	metformin 500mg tablets (almus pharmaceuticals ltd)
48401	6010203	Jan-13	other antidiabetic drugs	sitagliptin 50mg tablets
48533	6010203	Jan-13	other antidiabetic drugs	sitagliptin 25mg tablets
49108	6010101	Jan-13	short-acting insulins	novorapid penfill 100units/ml solution for injection 3ml cartridges (necessity supplies ltd)
49502	6010202	Jan-13	biguanides	glucophage sr 500mg tablets (mawdsley-brooks & company ltd)
49738	6010202	Jan-13	biguanides	metformin 1g modified-release tablets (a a h pharmaceuticals ltd)
49831	6010102	Jan-13	intermediate- and long-acting insulins	lantus 100units/ml solution for injection 3ml pre-filled solostar pen (necessity supplies ltd)
50087	6010203	Jan-13	other antidiabetic drugs	januvia 50mg tablets (merck sharp & dohme ltd)
50124	6010203	Jan-13	other antidiabetic drugs	januvia 25mg tablets (merck sharp & dohme ltd)
50570	6010202	Jan-13	biguanides	glucophage sr 500mg tablets (lexon (uk) ltd)
50633	6010102	Jan-13	intermediate- and long-acting insulins	lantus 100units/ml solution for injection 3ml cartridges (necessity supplies ltd)
50682	06010202/06010203	Jan-13	biguanides/other antidiabetic drugs	jentadueto 2.5mg/1000mg tablets (boehringer ingelheim ltd)

50691	0	Jan-13	unknown	human mixtard 20 penfill 100units/ml suspension for injection 1.5ml cartridges (novo nordisk ltd)
50821	6010202	Jan-13	biguanides	metformin 850mg tablets (pfizer ltd)
50970	6010202	Jan-13	biguanides	metformin 500mg tablets (bristol laboratories ltd)
51080	6010202	Jan-13	biguanides	metabet sr 1000mg tablets (actavis uk ltd)
51135	6010202	Jan-13	biguanides	metformin 500mg modified-release tablets (a a h pharmaceuticals ltd)
51527	6010202	Jan-13	biguanides	metformin 500mg tablets (boston healthcare ltd)
51743	6010101	Jan-13	short-acting insulins	novorapid penfill 100units/ml solution for injection 3ml cartridges (sigma pharmaceuticals plc)
51955	6010201	Jan-13	sulphonylureas	gliclazide 80mg tablets (accord healthcare ltd)
52203	6010203	Jan-13	other antidiabetic drugs	enyglid 0.5mg tablets (consilient health ltd)
52221	6010202	Jan-13	biguanides	diagemet xl 500mg tablets (genus pharmaceuticals ltd)
52442	6010202	Jan-13	biguanides	metformin 500mg tablets (pfizer ltd)
52445	06010202/06010203	Jan-13	biguanides/other antidiabetic drugs	linagliptin 2.5mg / metformin 1g tablets
52449	06010202/06010203	Jan-13	biguanides/other antidiabetic drugs	linagliptin 2.5mg / metformin 850mg tablets
52522	6010102	Jan-13	intermediate- and long-acting insulins	humalog mix50 kwikpen 100units/ml suspension for injection 3ml pre-filled pen (de pharmaceuticals)
52634	6010202	Jan-13	biguanides	glucophage sr 500mg tablets (de pharmaceuticals)
52722	0	Jan-13	unknown	human mixtard 30 penfill 100units/ml suspension for injection 1.5ml cartridges (novo nordisk ltd)
52748	6010102	Jan-13	intermediate- and long-acting insulins	insulatard penfill 100units/ml suspension for injection 3ml cartridges (waymade healthcare plc)
53118	6010101	Jan-13	short-acting insulins	novorapid flexpen 100units/ml solution for injection 3ml pre-filled pen (mawdsley-brooks & company ltd)
53251	6010101	Jan-13	short-acting insulins	novorapid penfill 100units/ml solution for injection 3ml cartridges (de pharmaceuticals)
53288	6010201	Jan-13	sulphonylureas	gliclazide 30mg modified-release tablets (a a h pharmaceuticals ltd)
53478	6010202	Jan-13	biguanides	metformin 500mg modified-release tablets (kent pharmaceuticals ltd)
53710	6010100	Feb-13	insulins	insulin human 500units/ml solution for injection 20ml vials
53774	6010202	Feb-13	biguanides	metabet sr 500mg tablets (actavis uk ltd)
53867	6010202	Feb-13	biguanides	metformin 500mg tablets (zentiva)
54150	06010202/06010203	Mar-13	biguanides/other antidiabetic drugs	jentadueto 2.5mg/850mg tablets (boehringer ingelheim ltd)
54182	6010203	Mar-13	other antidiabetic drugs	dapagliflozin 10mg tablets
54203	6010203	Mar-13	other antidiabetic drugs	forxiga 10mg tablets (astrazeneca uk ltd)
54265	6010203	Mar-13	other antidiabetic drugs	dapagliflozin 5mg tablets
54442	6010202	Apr-13	biguanides	metformin (roi) 1000mg tablet
54462	6010102	Apr-13	intermediate- and long-acting insulins	insulin biphasic isophane human emp 25:75; 100 units/ml injection
54480	6010203	Apr-13	other antidiabetic drugs	forxiga 5mg tablets (astrazeneca uk ltd)
54764	6010201	Apr-13	sulphonylureas	gliclazide 80mg tablets (arrow generics ltd)
54891	6010203	May-13	other antidiabetic drugs	saxagliptin 2.5mg / metformin 1g tablets
54898	6010202	May-13	biguanides	metformin 850mg tablets (almus pharmaceuticals ltd)
54973	6010203	May-13	other antidiabetic drugs	saxagliptin 2.5mg / metformin 850mg tablets
55234	6010102	May-13	intermediate- and long-acting insulins	tresiba flextouch 200units/ml solution for injection 3ml pre-filled pen (novo nordisk ltd)
55270	6010202	May-13	biguanides	duformin 500mg tablet (dumex ltd)
55413	6010203	May-13	other antidiabetic drugs	lixisenatide 20micrograms/0.2ml solution for injection 3ml pre-filled disposable devices
55459	6010203	Jun-13	other antidiabetic drugs	lixisenatide 10micrograms/0.2ml solution for injection 3ml pre-filled disposable devices
55462	6010102	Jun-13	intermediate- and long-acting insulins	tresiba flextouch 100units/ml solution for injection 3ml pre-filled pen (novo nordisk ltd)
55517	6010102	Jun-13	intermediate- and long-acting insulins	insulin isophane human 100units/ml suspension for injection 10ml vials
55603	6010101	Jun-13	short-acting insulins	humalog kwikpen 100units/ml solution for injection 3ml pre-filled pen (de pharmaceuticals)
55618	6010102	Jun-13	intermediate- and long-acting insulins	levemir flexpen 100units/ml solution for injection 3ml pre-filled pen (waymade healthcare plc)
55687	6010102	Jun-13	intermediate- and long-acting insulins	insulin degludec 100units/ml solution for injection 3ml pre-filled disposable devices
55711	6010202	Jun-13	biguanides	metformin 500mg tablets (alliance healthcare (distribution) ltd)
55723	6010203	Jun-13	other antidiabetic drugs	lixisenatide 10micrograms/0.2ml solution for injection 3ml pre-filled disposable devices and lixisenatide 20micrograms/0.2ml solution for injection 3ml pre-filled disposable devices
55728	6010203	Jun-13	other antidiabetic drugs	lyxumia 10micrograms/0.2ml solution for injection 3ml pre-filled pen (sanofi)
55729	6010203	Jun-13	other antidiabetic drugs	lyxumia 20micrograms/0.2ml solution for injection 3ml pre-filled pen (sanofi)
55739	6010202	Jun-13	biguanides	metformin 500mg tablets (tillomed laboratories ltd)
55767	6010203	Jun-13	other antidiabetic drugs	lyxumia 10micrograms/20micrograms treatment initiation pack (sanofi)
55862	6010201	Aug-13	sulphonylureas	gliclazide oral solution
55907	6010102	Aug-13	intermediate- and long-acting insulins	insulin degludec 100units/ml solution for injection 3ml cartridges

55910	6010102	Aug-13	intermediate- and long-acting insulins	tresiba penfill 100units/ml solution for injection 3ml cartridges (novo nordisk ltd)
56008	6010201	Aug-13	sulphonylureas	gliclazide 80mg tablets (almus pharmaceuticals ltd)
56115	0	Aug-13	unknown	human actrapid penfill 100units/ml solution for injection 1.5ml cartridges (novo nordisk ltd)
56208	6010203	Aug-13	other antidiabetic drugs	pioglitazone 15mg tablets (a a h pharmaceuticals ltd)
56376	06010201/06010203	Aug-13	sulphonylureas/other antidiabetic drugs	rosiglitazone 4mg with glimepiride 4mg tablet
56437	6010201	Aug-13	sulphonylureas	gliclazide 60mg modified-release tablets
56489	6010151	Aug-13	biphasic insulins	novomix 30 penfill 100units/ml suspension for injection 3ml cartridges (waymade healthcare plc)
56495	0	Aug-13	unknown	lantus 100units/ml solution for injection 3ml pre-filled optiset pen (waymade healthcare plc)
56502	0	Aug-13	unknown	actrapid penfill 100units/ml solution for injection 3ml cartridges (novo nordisk ltd)
56691	6010102	Aug-13	intermediate- and long-acting insulins	insulin degludec 200units/ml solution for injection 3ml pre-filled disposable devices
56831	6010203	Aug-13	other antidiabetic drugs	troglitazone 200mg tablet
56857	6010151	Aug-13	biphasic insulins	insulin isophane biphasic human 15/85 100units/ml suspension for injection 3ml cartridges
56965	6010203	Sep-13	other antidiabetic drugs	komboglyze 2.5mg/1000mg tablets (astrazeneca uk ltd)
57147	6010202	Sep-13	biguanides	bolamyn sr 1000mg tablets (teva uk ltd)
57457	6010202	Oct-13	biguanides	metformin 500mg tablets (aurobindo pharma ltd)
57529	6010101	Oct-13	short-acting insulins	humalog 100units/ml solution for injection 10ml vials (dowelhurst ltd)
57564	6010101	Oct-13	short-acting insulins	humalog kwikpen 100units/ml solution for injection 3ml pre-filled pen (waymade healthcare plc)
57601	6010201	Oct-13	sulphonylureas	daonil 5mg tablets (dowelhurst ltd)
57620	0	Oct-13	unknown	humulin m3 100units/ml suspension for injection 10ml vials (mawdsley-brooks & company ltd)
57622	6010102	Oct-13	intermediate- and long-acting insulins	humalog mix50 kwikpen 100units/ml suspension for injection 3ml pre-filled pen (waymade healthcare plc)
57659	6010203	Oct-13	other antidiabetic drugs	pioglitazone 30mg tablets (actavis uk ltd)
57830	6010201	Dec-13	sulphonylureas	gliclazide 30mg modified-release tablets (alliance healthcare (distribution) ltd)
58051	6010202	Dec-13	biguanides	metformin 500mg/5ml oral solution
58607	6010202	Jan-14	biguanides	metformin 500mg/5ml oral solution sugar free (zentiva)
58865	6010203	Feb-14	other antidiabetic drugs	komboglyze 2.5mg/850mg tablets (astrazeneca uk ltd)
58882	6010201	Feb-14	sulphonylureas	gliclazide 120mg/5ml oral suspension
59177	6010203	Mar-14	other antidiabetic drugs	alogliptin 25mg tablets
59385	6010203	Apr-14	other antidiabetic drugs	vipdomet 12.5mg/1000mg tablets (takeda uk ltd)
59500	6010102	Apr-14	intermediate- and long-acting insulins	insulin isophane human 100units/ml suspension for injection 5ml vials
59533	6010101	Apr-14	short-acting insulins	novorapid flexpen 100units/ml solution for injection 3ml pre-filled pen (sigma pharmaceuticals plc)
59620	6010202	Apr-14	biguanides	glucophage sr 500mg tablets (waymade healthcare plc)
59809	6010203	May-14	other antidiabetic drugs	alogliptin 6.25mg tablets
60012	6010202	May-14	biguanides	dapagliflozin 5mg / metformin 1g tablets
60066	0	May-14	unknown	invokana 100mg tablets (janssen-cilag ltd)
60073	6010203	Jun-14	other antidiabetic drugs	canagliflozin 100mg tablets
60074	6010202	Jun-14	biguanides	metformin 1g modified-release tablets (waymade healthcare plc)
60211	6010203	Jun-14	other antidiabetic drugs	canagliflozin 100mg tablets
60286	6010202	Jun-14	biguanides	metformin 500mg/5ml oral suspension
60328	6010203	Jul-14	other antidiabetic drugs	alogliptin 12.5mg tablets
60379	6010203	Jul-14	other antidiabetic drugs	invokana 300mg tablets (janssen-cilag ltd)
60386	6010203	Jul-14	other antidiabetic drugs	canagliflozin 300mg tablets
60430	6010203	Jul-14	other antidiabetic drugs	invokana 100mg tablets (janssen-cilag ltd)
60495	6010201	Jul-14	sulphonylureas	gliclazide 80mg tablets (teva uk ltd)
60497	6010203	Jul-14	other antidiabetic drugs	alogliptin 12.5mg / metformin 1g tablets
60643	6010202	Jul-14	biguanides	xigduo 5mg/1000mg tablets (astrazeneca uk ltd)
60681	6010203	Aug-14	other antidiabetic drugs	vipidia 12.5mg tablets (takeda uk ltd)
60682	6010203	Aug-14	other antidiabetic drugs	vipidia 25mg tablets (takeda uk ltd)
60933	6010151	Aug-14	biphasic insulins	humulin m3 100units/ml suspension for injection 10ml vials (sigma pharmaceuticals plc)
60938	0	Aug-14	unknown	mixtard 30 100units/ml suspension for injection 10ml vials (waymade healthcare plc)
60951	6010100	Aug-14	insulins	insulin human 100units/ml solution for injection 10ml vials
60968	6010202	Aug-14	biguanides	metformin 500mg modified-release tablets (actavis uk ltd)
61043	6010202	Sep-14	biguanides	sukkarto sr 1000mg tablets (morningside healthcare ltd)
61311	6010201	Oct-14	sulphonylureas	glimepiride 4mg tablets (sigma pharmaceuticals plc)
61559	6010202	Nov-14	biguanides	sukkarto sr 500mg tablets (morningside healthcare ltd)
61756	6010203	Dec-14	other antidiabetic drugs	empagliflozin 10mg tablets

61845	6010101	Dec-14	short-acting insulins	novorapid pumpcart 100units/ml solution for injection 1.6ml cartridges (novo nordisk ltd)
61925	6010203	Jan-15	other antidiabetic drugs	novonorm 500microgram tablets (waymade healthcare plc)
61957	6010201	Jan-15	sulphonylureas	gliclazide 40mg tablets (a a h pharmaceuticals ltd)
62014	6010201	Jan-15	sulphonylureas	glimepiride 2mg tablets (accord healthcare ltd)
62034	6010201	Jan-15	sulphonylureas	laaglyda mr 60mg tablets (consilient health ltd)
62144	6010202	Jan-15	biguanides	metformin 500mg modified-release tablets (de pharmaceuticals)
62172	6010203	Jan-15	other antidiabetic drugs	empagliflozin 25mg tablets
62180	6010101	Jan-15	short-acting insulins	insulin aspart 100units/ml solution for injection 1.6ml cartridges
62265	6010202	Jan-15	biguanides	metformin 500mg modified-release tablets (mawdsley-brooks & company ltd)
62276	6010100	Jan-15	insulins	humulin r 500units/ml solution for injection 20ml vials (imported (united states))
62326	6010203	Feb-15	other antidiabetic drugs	vipidia 6.25mg tablets (takeda uk ltd)
62426	6010203	Feb-15	other antidiabetic drugs	pioglitazone 30mg tablets (accord healthcare ltd)
62605	6010202	Mar-15	biguanides	metformin 850mg tablets (kent pharmaceuticals ltd)
62661	6010203	Mar-15	other antidiabetic drugs	bydureon 2mg powder and solvent for prolonged-release suspension for injection pre-filled pen (astrazeneca uk ltd)
62760	6010203	Apr-15	other antidiabetic drugs	jardiance 10mg tablets (boehringer ingelheim ltd)
62824	6010202	Apr-15	biguanides	metformin 1g modified-release tablets (actavis uk ltd)
62899	06010102/06010203	Apr-15	intermediate- and long-acting insulins/other antidiabetic drugs	xultophy 100units/ml / 3.6mg/ml solution for injection 3ml pre-filled pen (novo nordisk ltd)
62904	6010203	Apr-15	other antidiabetic drugs	exenatide 2mg powder and solvent for prolonged-release suspension for injection pre-filled disposable devices
63031	6010202	Apr-15	biguanides	dapagliflozin 5mg / metformin 850mg tablets
63045	6010202	Apr-15	biguanides	metformin 850mg tablets (relonchem ltd)
63046	6010203	Apr-15	other antidiabetic drugs	pioglitazone 45mg tablets (a a h pharmaceuticals ltd)
63048	6010201	Apr-15	sulphonylureas	gliclazide 80mg tablets (alliance healthcare (distribution) ltd)
63107	6010203	May-15	other antidiabetic drugs	pioglitazone 45mg tablets (waymade healthcare plc)
63131	6010201	May-15	sulphonylureas	ziclaseg 30mg modified-release tablets (lupin (europe) ltd)
63307	6010202	Jun-15	biguanides	metformin 1g/5ml oral solution
63336	6010203	Jun-15	other antidiabetic drugs	trulicity 1.5mg/0.5ml solution for injection pre-filled pen (eli lilly and company ltd)
63401	6010203	Jun-15	other antidiabetic drugs	trulicity 0.75mg/0.5ml solution for injection pre-filled pen (eli lilly and company ltd)
63421	6010203	Jun-15	other antidiabetic drugs	pioglitazone 30mg tablets (teva uk ltd)
63464	6010101	Jun-15	short-acting insulins	humalog kwikpen 200units/ml solution for injection 3ml pre-filled pen (eli lilly and company ltd)
63516	6010203	Jun-15	other antidiabetic drugs	forxiga 10mg tablets (waymade healthcare plc)
63562	06010102/06010203	Jul-15	intermediate- and long-acting insulins/other antidiabetic drugs	insulin degludec 100units/ml / liraglutide 3.6mg/ml solution for injection 3ml pre-filled disposable devices
63679	06010101/06010300	Jul-15	short-acting insulins/diabetic ketoacidosis	hypurin soluble 100iu/ml injection (c p pharmaceuticals ltd)
63785	6010203	Jul-15	other antidiabetic drugs	dulaglutide 0.75mg/0.5ml solution for injection pre-filled disposable devices
63823	6010203	Jul-15	other antidiabetic drugs	dulaglutide 1.5mg/0.5ml solution for injection pre-filled disposable devices
63929	6010203	Aug-15	other antidiabetic drugs	canagliflozin 50mg / metformin 1g tablets
64217	6010203	Sep-15	other antidiabetic drugs	jardiance 25mg tablets (boehringer ingelheim ltd)
64354	6010102	Oct-15	intermediate- and long-acting insulins	toujeo 300units/ml solution for injection 1.5ml pre-filled solostar pen (sanofi)
64460	6010102	Oct-15	intermediate- and long-acting insulins	insulin glargine 300units/ml solution for injection 1.5ml pre-filled disposable devices
64622	6010203	Nov-15	other antidiabetic drugs	bydureon 2mg powder and solvent for prolonged-release suspension for injection vials (lexon (uk) ltd)
64723	6010102	Nov-15	intermediate- and long-acting insulins	abasaglar kwikpen 100units/ml solution for injection 3ml pre-filled pen (eli lilly and company ltd)
64743	6010203	Nov-15	other antidiabetic drugs	canagliflozin 50mg / metformin 850mg tablets
64900	6010203	Dec-15	other antidiabetic drugs	glidipion 30mg tablets (actavis uk ltd)
64939	6010202	Dec-15	biguanides	glucient sr 1000mg tablets (consilient health ltd)
64987	6010102	Dec-15	intermediate- and long-acting insulins	abasaglar 100units/ml solution for injection 3ml cartridges (eli lilly and company ltd)
65057	06010202/06010203	Dec-15	biguanides/other antidiabetic drugs	empagliflozin 5mg / metformin 1g tablets
65059	6010202	Dec-15	biguanides	xigduo 5mg/850mg tablets (astrazeneca uk ltd)
65066	06010202/06010203	Dec-15	biguanides/other antidiabetic drugs	empagliflozin 12.5mg / metformin 1g tablets
65083	06010202/06010203	Dec-15	biguanides/other antidiabetic drugs	synjardy 5mg/1000mg tablets (boehringer ingelheim ltd)
65344	06010202/06010203	Jan-16	biguanides/other antidiabetic drugs	empagliflozin 5mg / metformin 850mg tablets
65562	6010203	Feb-16	other antidiabetic drugs	pioglitazone 30mg tablets (alliance healthcare (distribution) ltd)
65563	6010203	Feb-16	other antidiabetic drugs	pioglitazone 15mg tablets (alliance healthcare (distribution) ltd)
65694	6010202	Mar-16	biguanides	metformin 500mg modified-release tablets (waymade healthcare plc)
65923	6010202	Apr-16	biguanides	metformin 1g modified-release tablets (mawdsley-brooks & company ltd)
66008	06010202/06010203	Apr-16	biguanides/other antidiabetic drugs	synjardy 12.5mg/1000mg tablets (boehringer ingelheim ltd)
66136	6010202	May-16	biguanides	glucophage sr 1000mg tablets (waymade healthcare plc)

66316	6010102	Jun-16	intermediate- and long-acting insulins	lantus 100units/ml solution for injection 10ml vials (mawdsley-brooks & company ltd)
66335	6010102	Jun-16	intermediate- and long-acting insulins	insulin biphasic isophane porcine 50:50; 100 units/ml injection
66399	6010201	Jun-16	sulphonylureas	glimepiride 2mg tablets (a a h pharmaceuticals ltd)
66854	6010203	Jul-16	other antidiabetic drugs	vokanamet 50mg/1000mg tablets (janssen-cilag ltd)
66855	06010202/06010203	Jul-16	biguanides/other antidiabetic drugs	empagliflozin 12.5mg / metformin 850mg tablets
67056	6010201	Aug-16	sulphonylureas	amaryl 1mg tablets (lexon (uk) ltd)
67230	6010102	Aug-16	intermediate- and long-acting insulins	lantus 100units/ml solution for injection 3ml pre-filled solostar pen (waymade healthcare plc)
67231	0	Aug-16	unknown	novorapid flexpen 100units/ml solution for injection 3ml pre-filled pen (dowelhurst ltd)
67266	0	Aug-16	unknown	mixtard 50 penfill 100units/ml suspension for injection 3ml cartridges (waymade healthcare plc)
67267	0	Aug-16	unknown	mixtard 30 innolet 100units/ml suspension for injection 3ml pre-filled pen (waymade healthcare plc)
67279	0	Aug-16	unknown	pork insulatard 100units/ml suspension for injection 10ml vials (waymade healthcare plc)
67313	6010101	Aug-16	short-acting insulins	novorapid 100units/ml solution for injection 10ml vials (sigma pharmaceuticals plc)
67324	6010151	Aug-16	biphasic insulins	humulin m3 100units/ml suspension for injection 3ml cartridges (waymade healthcare plc)
67429	6010100	Sep-16	insulins	insulin human 100units/ml solution for injection 3.15ml cartridges
67781	6010201	Oct-16	sulphonylureas	gliclazide 80mg tablets (milpharm ltd)
68031	6010102	Oct-16	intermediate- and long-acting insulins	humalog mix25 kwikpen 100units/ml suspension for injection 3ml pre-filled pen (sigma pharmaceuticals plc)
68203	6010202	Nov-16	biguanides	metformin 500mg modified-release tablets (almus pharmaceuticals ltd)
68214	6010202	Nov-16	biguanides	metformin 500mg/5ml oral solution sugar free (a a h pharmaceuticals ltd)
68258	6010203	Nov-16	other antidiabetic drugs	alogliptin 25mg tablets (colorama pharmaceuticals ltd)
68289	6010201	Dec-16	sulphonylureas	glimepiride 4mg tablets (waymade healthcare plc)
68389	6010202	Dec-16	biguanides	metformin 500mg/5ml oral solution sugar free (pinewood healthcare)
68415	6010201	Dec-16	sulphonylureas	gliclazide 30mg modified-release tablets (phoenix healthcare distribution ltd)
68589	6010202	Jan-17	biguanides	metformin 1g/5ml oral solution sugar free
68636	6010202	Jan-17	biguanides	metformin 850mg/5ml oral solution sugar free
68675	6010201	Jan-17	sulphonylureas	glimepiride 4mg tablets (somex pharma)
